# Supplementary material for: Genetic incorporation of non-canonical amino acid photocrosslinkers in Neisseria meningitidis: New method provides insights into the physiological function of the function-unknown NMB1345 protein
Source: PLoS One. 2020 Aug 31;15(8):e0237883. doi: 10.1371/journal.pone.0237883 (PMC7458321; doi:10.1371/journal.pone.0237883)
Supplement: S1 Fig — (GenBank Accession No LC511747). Lysine (K) residues are shown in red and the K residues replaced with amber codons in this study are indicated in bold red. Positions of K residues are shown as blue numbers. The grey bar indicates the putative hydrophobic region identified by SOSUI [90], which region were deleted to construct the PamA recombinants in E. coli in this study. (PDF) [file pone.0237883.s001.pdf]

```
ATGAAAAAACCTTTGATTTCGGTTGCGGCAGCATTGCTCGGCGTTGCTTTGGGCACGCCTTATTATTTGGGTGTCAAAGCCGAAGAAAGCTTGACGCAGCAGCAAAAAATATTGCAGGAA
M K K P L I S V A A A L L G V A L G T P Y Y L G V K A E E S L T Q Q Q K I L Q E

GCGGGCTTCTTGACCGTCGAATCGCACCAATATGAGCGCGGCTGGTTTACCTCTACGGAAACGACGGTCATCCGCTTGAAACCGGAGTTGCTGAATAATGCCCGGAAATACCTGCCGGAC
A G F L T V E S H Q Y E R G W F T S T E T T V I R L K P E L L N N A R K Y L P D

AACCTGAAAACAGTGTTGGAACAGCCGGTTACGCTGGTAAACCATATCACGCACGGCCCTTTCGCCGGCGGATTCGGCACGCAGGCGTACATTGAAACCGAGTTCAAATACGCGCCTGAA
N L K T V L E Q P V T L V N H I T H G P F A G G F G T Q A Y I E T E F K Y A P E

ACGGAAAAAGTTTTGGAACGCTTTTTTGGGAAACAAGCTCCGGTTTCCCTTGCCAATACCGTTTATTTTAACGGCAGCGGTAAAATGGAAGTCAGTGTTCCCGCCTTCGATTATGAAGAG
T E K V L E R F F G K Q A P V S L A N T V Y F N G S G K M E V S V P A F D Y E E
                                                       148

CTGTCGGGCATCAGGCTGCATTGGGAAGGATTGACGGGAAAAACGGTTTATCAAAAAGGTTTCAAAAGCTACCGCAACAGCTATGATGCGCCCTTGTTCAAAATCAAGCTGGCAGACAAA
L S G I R L H W E G L T G K T V Y Q K G F K S Y R N S Y D A P L F K I K L A D K
                          174         179   182                           194

GGCGATGCCGCGTTTGAAAAAGCGCATTTCGATTCGGAAACTTCAGACGGTATCAATCCGCTTGCTTTGGGCAGCAGCAATCTGACCTTGGAAAAATTCTCCCTAGAATGGAAAGAGGGT
G D A A F E K A H F D S E T S D G I N P L A L G S S N L T L E K F S L E W K E G
            208                                             232

GTCGATTACAACGTCAAGTTAAACGAACTGGTCAATCTTGTTACCGATTTGCAGATTGGCGCGTTTATCAATCCCAACGGCAGCATCGCACCTTCCAAAATCGAAGTCGGCAAACTGGCT
V D Y N V K L N E L V N L V T D L Q I G A F I N P N G S I A P S K I E V G K L A
          246                                                 273         278

TTTTCAACCAAGACCGGGGAATCAGGCTCATTTATCAACAGTGAAGGGCAGTTCCGTTTTGACACGCTGGTGTACGGCAATGAAAAATACGGCCCGCTGGACATCCATATCGCCGCCGAA
F S T K T G E S G S F I N S E G Q F R F D T L V Y G N E K Y G P L D I H I A A E
      284                                               309

CATCTCGATGCTTCTGCCTTAACCGTATTGAAACGTAAGTTTGCACAAATTTCCGCCAAAAAAATGACCGAGGAACAAATCCGCAATGATTTGATTGCCGCCGTCAAAGGCGAGGCTTCC
H L D A S A L T V L K R K F A Q I S A K K M T E E Q I R N D L I A A V K G E A S
                    331                 341                             356

GGACTGTTCACCAACAATCCCGTATTGGACATTAAAACTTTTCGTTTCACACAGCCGTCGGGAAAAATCGATGTGGGTGGAAAAATCATGTTTAAAGACATGAAGAAGGAAGATTTGAAT
G L F T N N P V L D I K T F R F T Q P S G K I D V G G K I M F K D M K K E D L N
                      371                 382         382         395

CAATTGGGTTTGATGCTGAAGAAAACCGAAGCCGACATCAGAATGAGTATTCCCCAAAAAATGCTGGAAGACTTGGCGGTCAGTCAAGCAGGCAATATTTTCAGCGTCAATGCCGAAGAT
Q L G L M L K K T E A D I R M S I P Q K M L E D L A V S Q A G N I F S V N A E D
            407 408                     420

GAGGCGGAAGGCAGGGCAAGTCTTGACGACATCAACGAGACCTTGCGCCTGATGGTGGACAGTACGGTTCAGAGTATGGCAAGGGAAAAATATCTGACTTTGAACGGCGACCAGATTGAT
E A E G R A S L D D I N E T L R L M V D S T V Q S M A R E K Y L T L N G D Q I D
                                                         470

ACTGCCATTTCTCTGAAAAACAATCAGTTGAAATTGAACGGTAAAACGTTGCAAAACGAACCGGAGCCGGATTTTGATGAAGGCGGTATGGTTTCAGAGCCGCAGCAGTAA
T A I S L K N N Q L K L N G K T L Q N E P E P D F D E G G M V S E P Q Q
          486       491     495
```
