# Supplementary figures and images for: Genetic incorporation of non-canonical amino acid photocrosslinkers in Neisseria meningitidis: New method provides insights into the physiological function of the function-unknown NMB1345 protein
Source: PLoS One. 2020 Aug 31;15(8):e0237883. doi: 10.1371/journal.pone.0237883 (PMC7458321; doi:10.1371/journal.pone.0237883)

**A**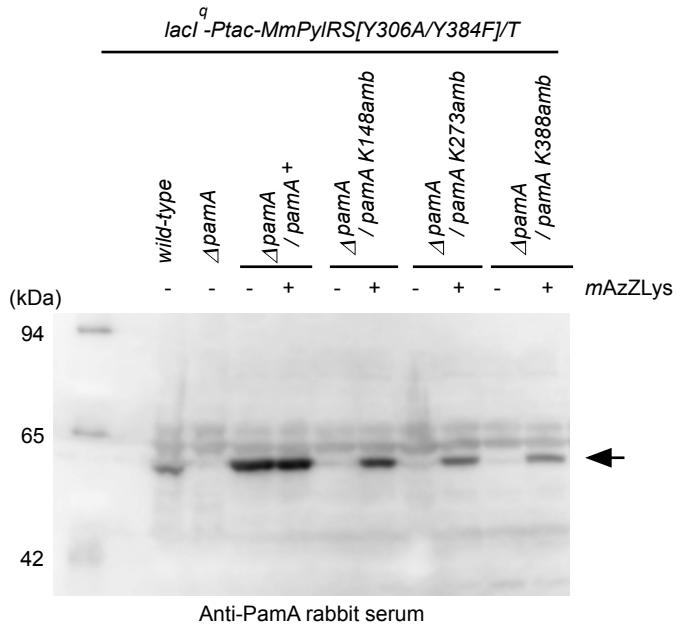**B**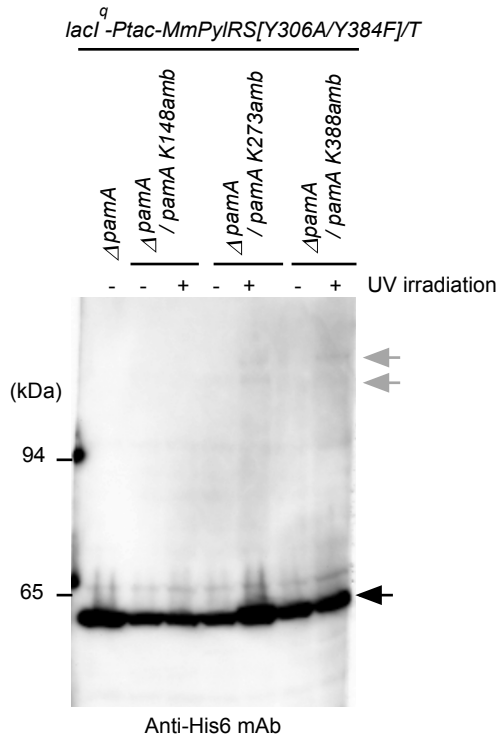

Supplement: S2 Fig — (A) Detection of PamA K148amb, K273 and K388amb expressed by pyrrolysine-based amber suppression with mAzZLys in N. meningitidis. Bacteria grown on one-fourth of a GC agar plate in the presence or absence of 0.3 mM mAzZLys and extracts equivalent to OD600 of 0.1 were analyzed by Western blotting with anti-PamA rabbit serum. +/- indicates the presence or absence of mAzZLys. The black arrow shows the full-length PamA protein. (B) Detection of complexes containing PamA K(mAzZLys) crosslinked to an endogenous protein in N. meningitidis. Bacteria grown on one-fourth of a GC agar plate in the presence of 0.3 mM mAzZLys were crosslinked by UV light irradiation, and mixed with SDS buffer. Aliquots were analyzed by Western blotting with an anti-His6 mAb. +/- indicates presence or absence of mAzZLys (upper), treatment or nontreatment with UV irradiation (lower). Black and grey arrows show the full-length PamA protein and putative complexes corresponding to PamA K(mAzZLys) crosslinked to an endogenous protein, respectively. (PDF) [file pone.0237883.s002.pdf]

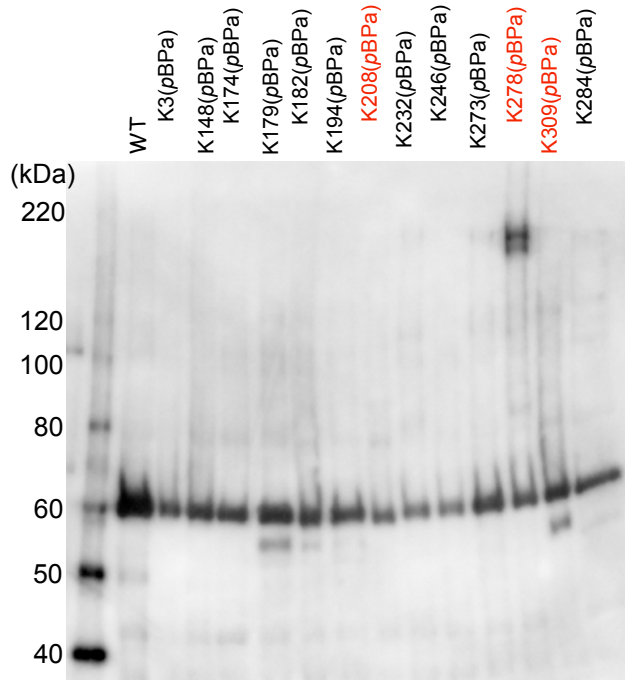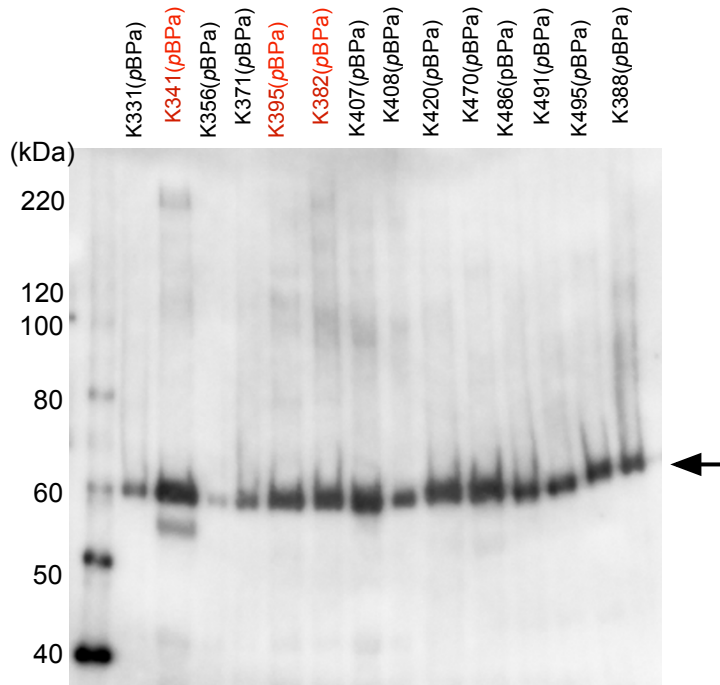

Supplement: S3 Fig — UV crosslinking of 27 PamA K-amb mutants expressed by pyrrolysine-based amber suppression with pBPa in ΔpamA N. meningitidis. The black arrow shows the full-length PamA K-amb protein expressed by pyrrolysine-based amber suppression with pBPa. PamA K-amb mutants shown in red were subjected to a more detailed analysis in Fig 4. (PDF) [file pone.0237883.s003.pdf]

**A**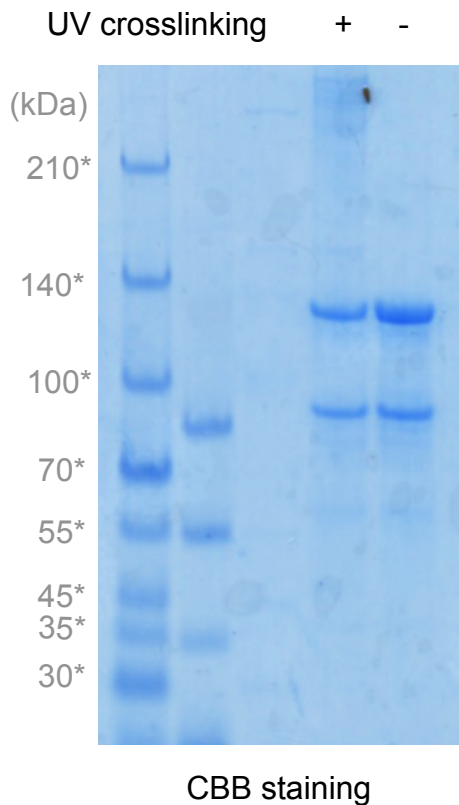**B**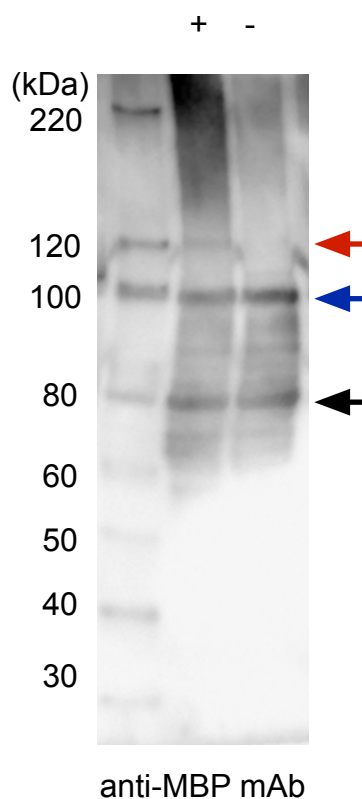**C**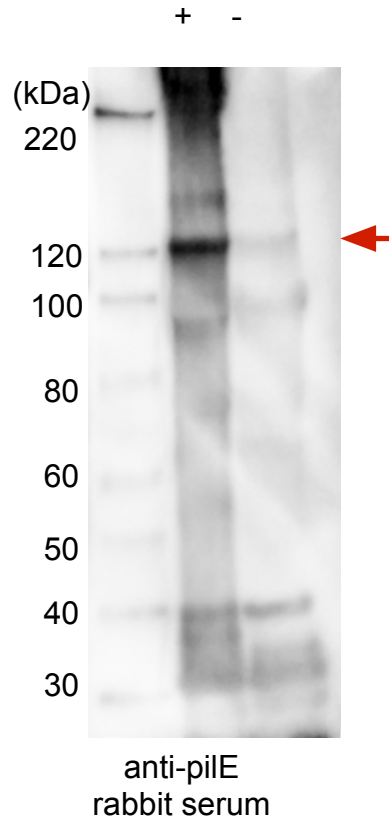**D**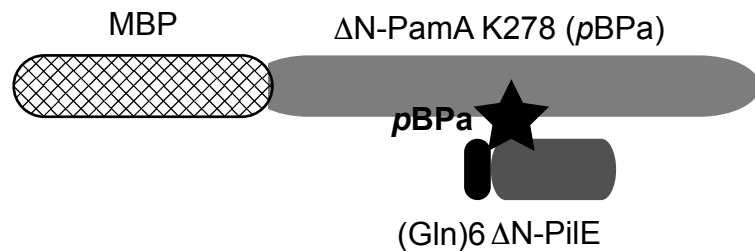

Supplement: S6 Fig — Recombinant proteins crosslinked in E. coli were purified with amylose resin and concentrated to 500 μl as described in the Materials and Methods. Aliquots (approximately 1 μl) were fractionated by SDS-PAGE and analyzed by staining with a CBB staining kit (A), and Western blotting with anti-MBP (B) and anti-His6 (C) mAbs. Prestained molecular mass standards are shown by grey numbers with asterisks, since the apparent molecular masses were different from the actual masses due to abnormal mobility in the gel. +/- indicates treatment or non-treatment with UV irradiation, respectively. Black, blue and red arrows indicate PamA ΔN-K278amb (no pyrrolysine-based amber suppression with pBPa), PamA ΔN-K278(pBPa)-Strep2-His6 (full-length) and the complex crosslinked between PamA K278(pBPa)-Strep2-His6 and ΔN-Gln6-PilE, respectively. (D) Schematic figure of the crosslinking between PamA ΔN-K278(pBPa)-Strep2-His6 and Gln6-ΔN-PilE recombinant proteins in E. coli. (PDF) [file pone.0237883.s006.pdf]

**A**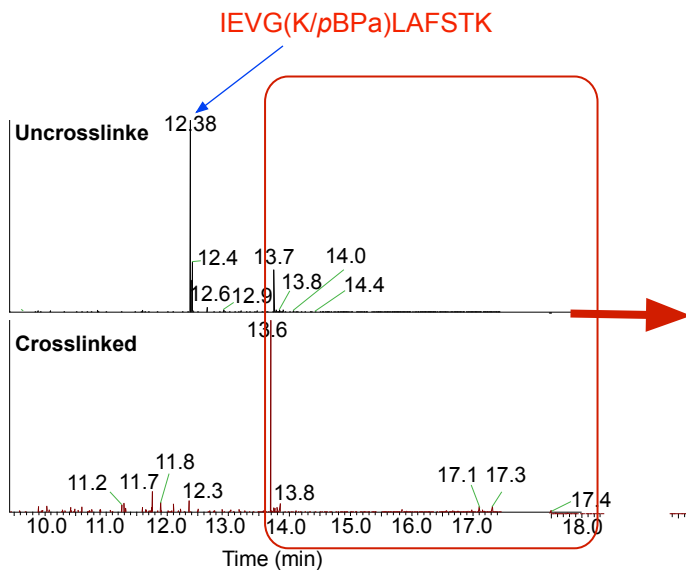**B**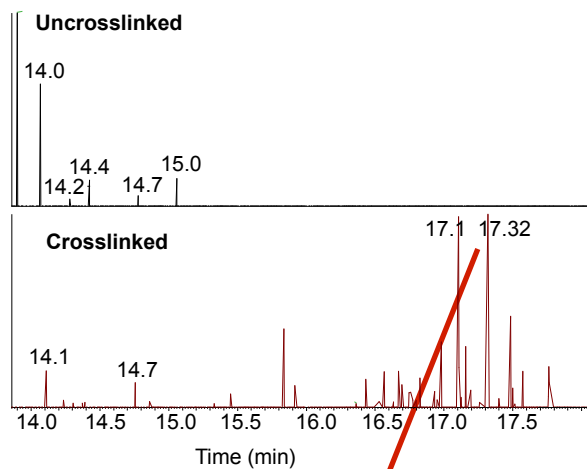**C**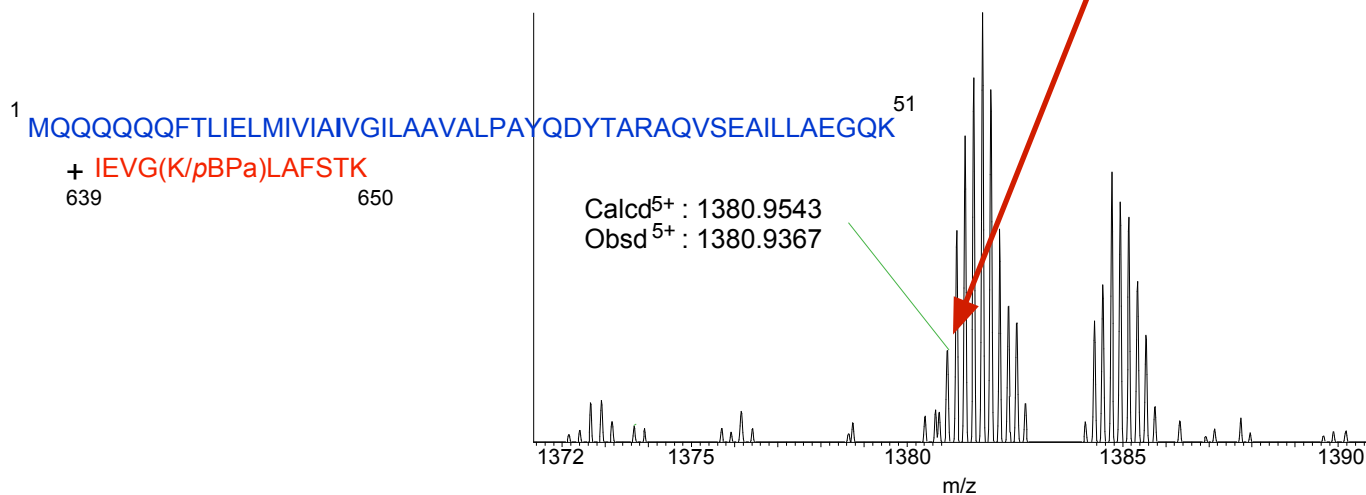

Supplement: S7 Fig — (A) Liquid chromatogram of uncrosslinked MBP-ΔN-PamA K278(pBPa) and the Gln6-ΔN-PilE protein (upper), and the complex crosslinked between MBP-ΔN-PamA K278(pBPa) and Gln6-ΔN-PilE (lower). The fraction eluted at 12.38 min from the uncrosslinked sample contained the peptide IEVGK(pBPa)LAFSTK corresponding to position 639 to 650 of MBP-ΔN-PamA K278(pBPa) (positions 274 to 284 for native PamA), determined by an MS analysis. (B) Enlarged liquid chromatogram after 14 min of elution. The fraction eluted at 17.32 min from the crosslinked sample contained the peptide MQQQQQQFTLIELMIVIAIVGILAAVALPAYQDYTARAQVSEAILLAEGQK, corresponding to the first 51 amino acids of Gln6-ΔN-PilE at the N terminus, which was crosslinked to the peptide IEVGK(pBPa)LAFSTK from the MBP-ΔN-PamA K278(pBPa) protein. (C) MS spectrum of the LC fraction eluted at 17.32 min that contained the cross-linked peptide between MQQQQQQFTLIELMIVIAIVGILAAVALPAYQDYTARAQVSEAILLAEGQK from Gln6-ΔN-PilE and IEVGK(pBPa)LAFSTK from MBP-ΔN-PamA K278(pBPa)(obsd:1380.9367 [M+H]5+). (PDF) [file pone.0237883.s007.pdf]

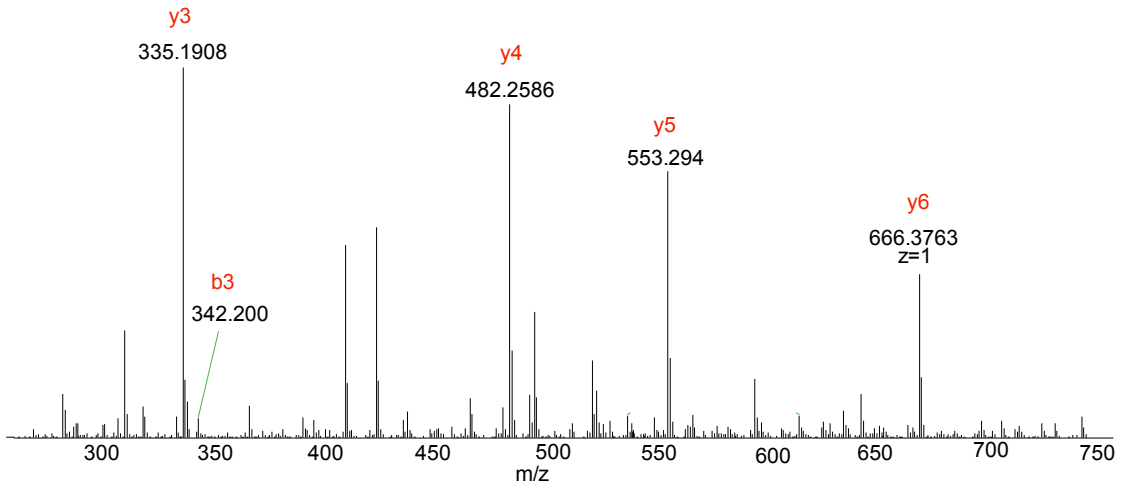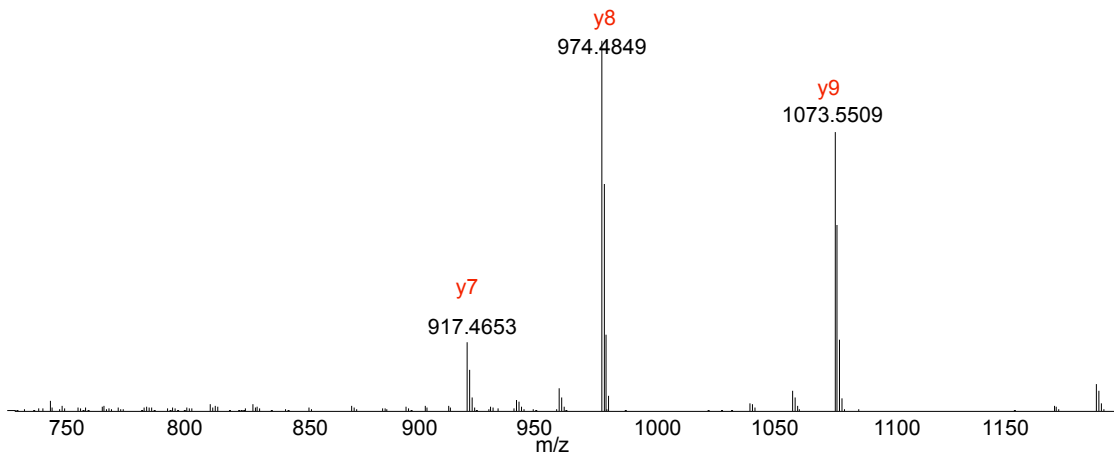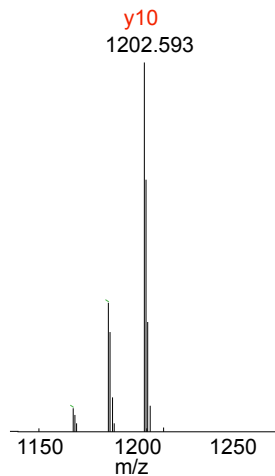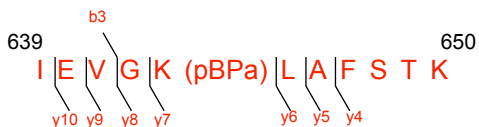

Supplement: S8 Fig — The sequence was read from the annotated b and y ion series; b3, y4, y5, y6, y7, y8, y9, and y10 ions were observed. (PDF) [file pone.0237883.s008.pdf]

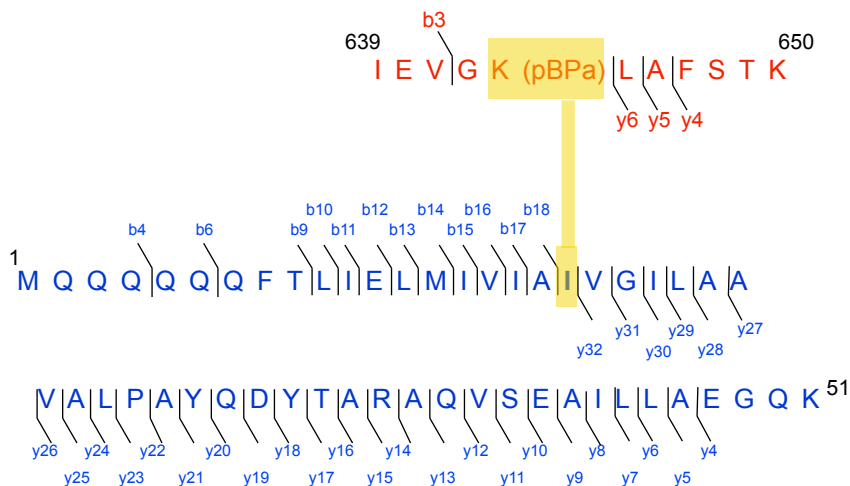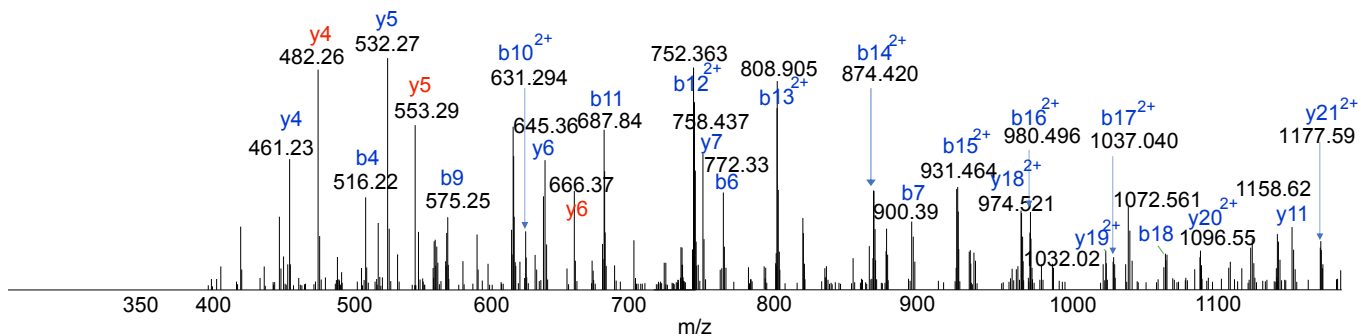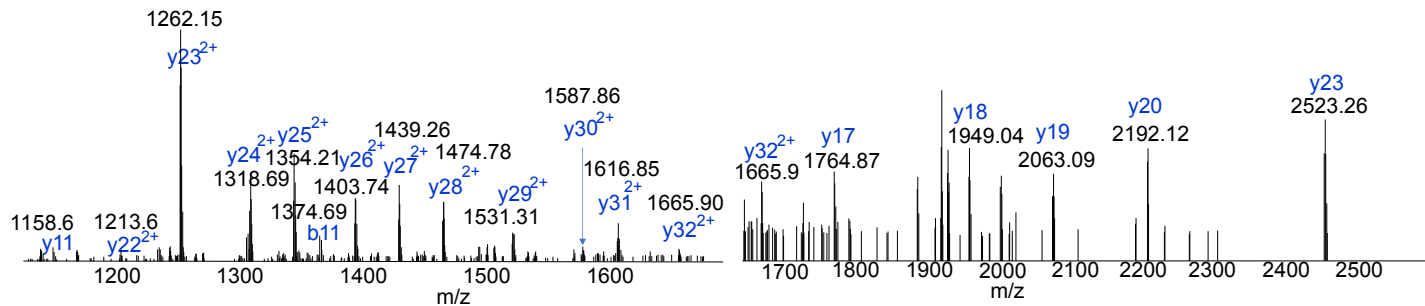

Supplement: S9 Fig — The sequence was read from the annotated b and y ion series; b3, y4, y5, y6, y7, y8, y9, and y10 ions of the peptide IEVGK(pBPa)LAFSTK from MBP-ΔN-PamA K278(pBPa) (shown in red), and b3, b6, b9, b10, b11, b12, b13, b14, b15, b16, b17, b18, y4, y5, y6, y7, y8, y9, y10, y11, y12, y13, y14, y15, y16, y17, y18, y19, y20, y21, y22, y23, y24, y25, y26, y27, y28, y29, y30, y31, and y32 ions of the peptide MQQQQQQFTLIELMIVIAIVGILAAVALPAYQDYTARAQVSEAILLAEGQK from Gln6-ΔN-PilE (shown in blue) were observed. (PDF) [file pone.0237883.s009.pdf]

**A**

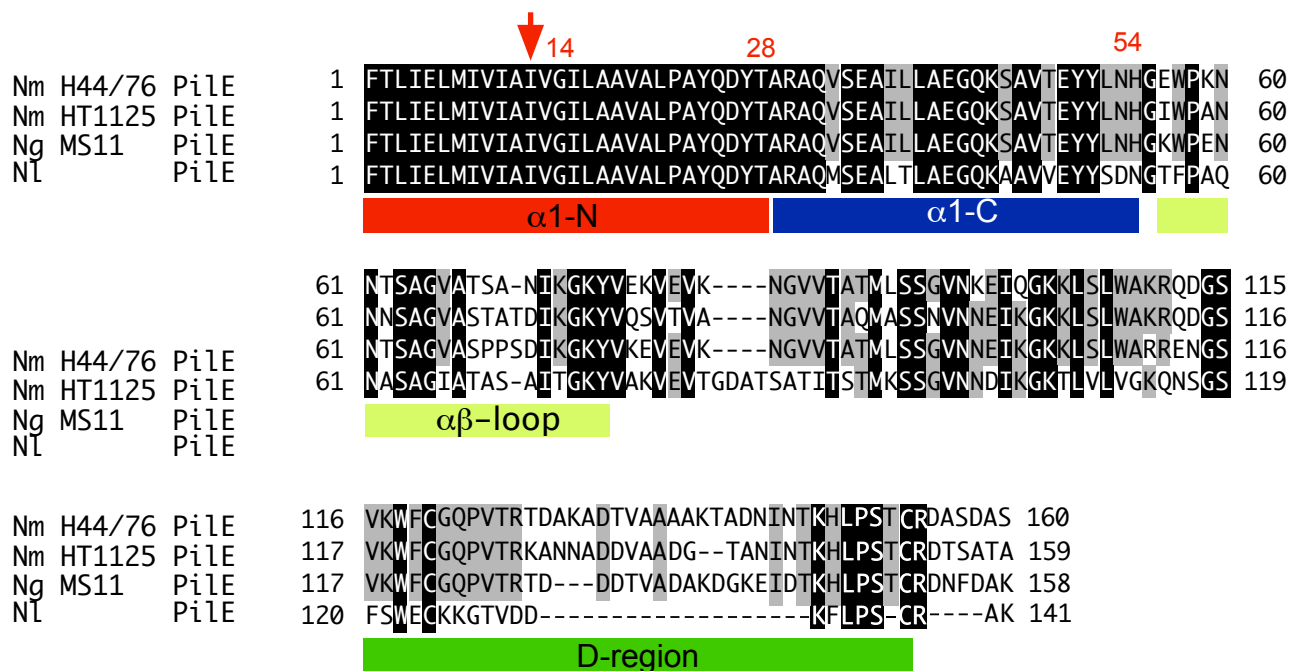

**B**

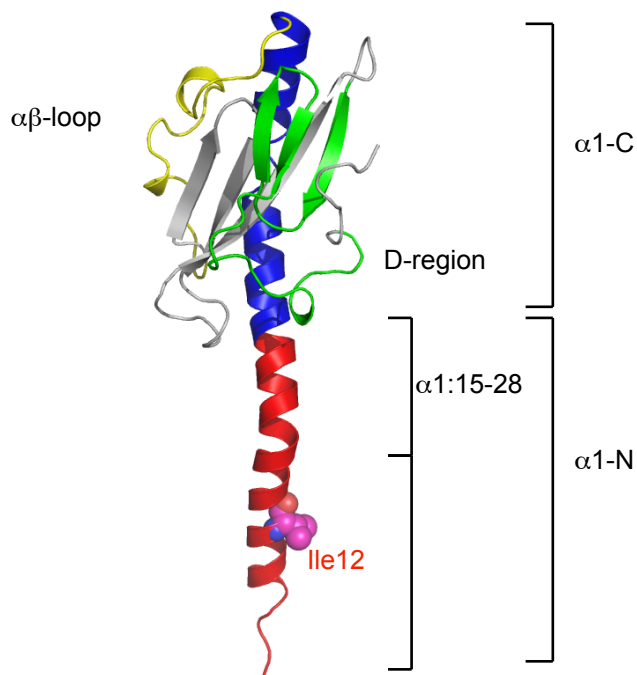

Supplement: S10 Fig — (A) Amino acid sequence alignment of PilE proteins from N. meningitidis (Nm), N. gonorrhoeae (Ng) and N. lactamica (Nl). The N-terminal half of the α-helix (α1-N) responsible for pilin assembly is shown by red (residues 1–14; α1:1–14) and cyan (residues 15–28; α1:15–28) bars, respectively. The αβ-loop, protruding from the globular domain to form a ridge on the subunit surface is indicated as a yellow bar. The D-region containing the hypervariable loop that protrudes as a second ridge on the globular domain is indicated as a light green bar. Red numbers indicate the positions of amino acids at which the functions of α1-N are divided. Ile at position 12 is indicated by a red vertical arrow. N. meningitidis HT1125 PilE (DDBJ accession no. AB698857), N. meningitidis H44/76 PilE (GenBank accession no. CP002420), N. gonorrhoeae MS11 PilE (EMBL accession no. CAI08338) and N. lactamica NLA_1780 type IV pilus assembly protein PilA (GenBank accession no. FN995097). (B) X-ray crystal structure of gonococcal PilE [91]. α1:1–14, α1:15–28, αβ-loop and D-region are shown in red, cyan, yellow and light green, respectively (the colors in B correspond to those in A). Ile at position 12 is indicated as a space-filling molecule. (PDF) [file pone.0237883.s010.pdf]

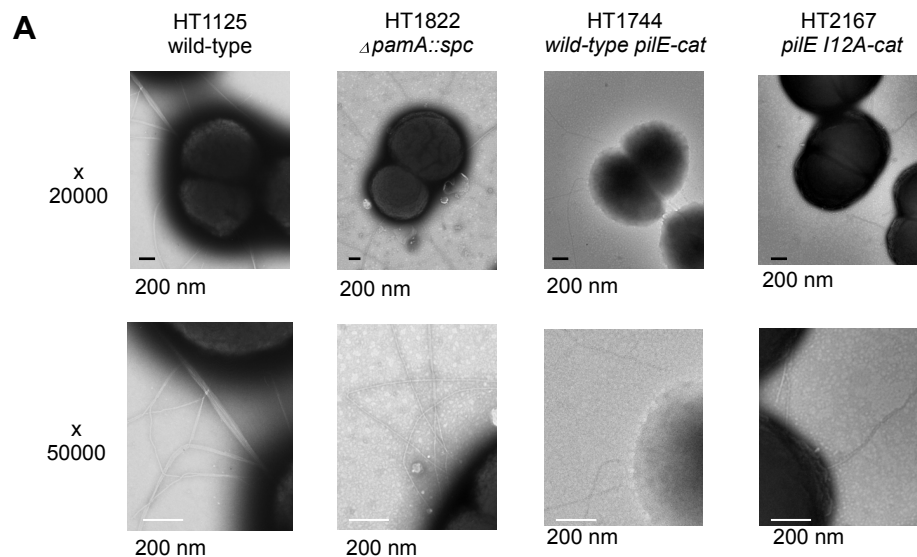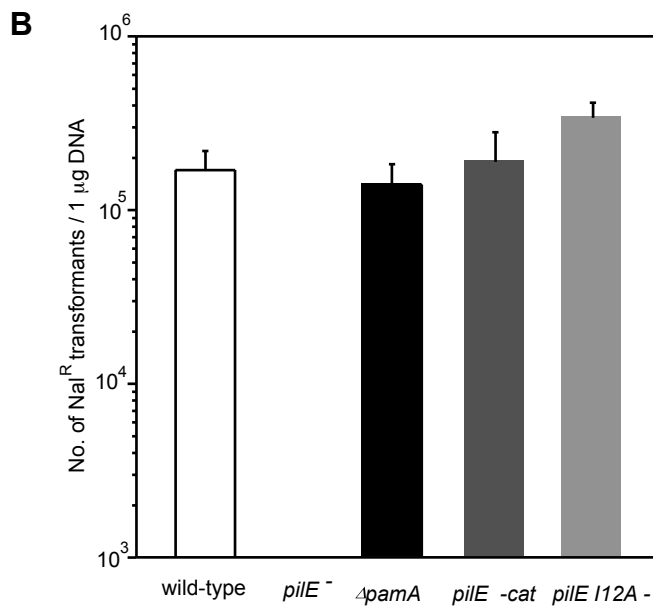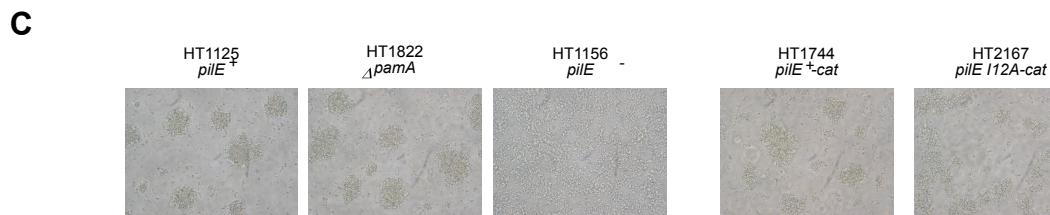

Supplement: S11 Fig — (A) Electron micrographs showing piliation of N. meningitidis strains HT1125 (wild-type), HT1822 (ΔpamA), HT1744 (pilE+-cat), and HT2167 (pilE I12A-cat). Upper and lower panels show magnifications of 20,000 and 50,000, respectively. Scale bars shown in black (upper) and white (lower) represent 200 nm. (B) Quantification of the competence for DNA transformation in N. meningitidis wild-type, and pilE-, ΔpamA, pilE+-cat and pilE I12A-cat mutants. Equivalent numbers of recipient cells were transformed using 0.5 μg of chromosomal DNA purified from the NalR (nalidixic acid resistant) strain HT1001, and NalR transformants were counted. Results are expressed as numbers of NalR transformants per 1 μg DNA and ± standard deviation from at least 8 independent experiments. (C) Aggregation as assessed by phase-contrast microscopy. Aggregates of N. meningitidis strains HT1125 (wild-type), HT1156 (pilE-), HT1822 (ΔpamA), HT1744 (pilE+-cat) and HT2167 (pilE I12A-cat) were observed after 4 hours of incubation in RPMI containing 10% fetal bovine serum. (PDF) [file pone.0237883.s011.pdf]

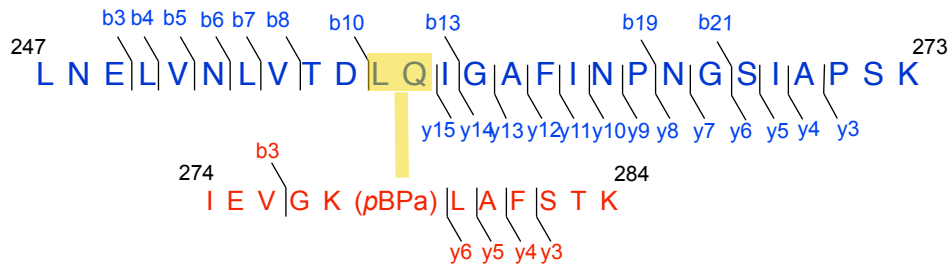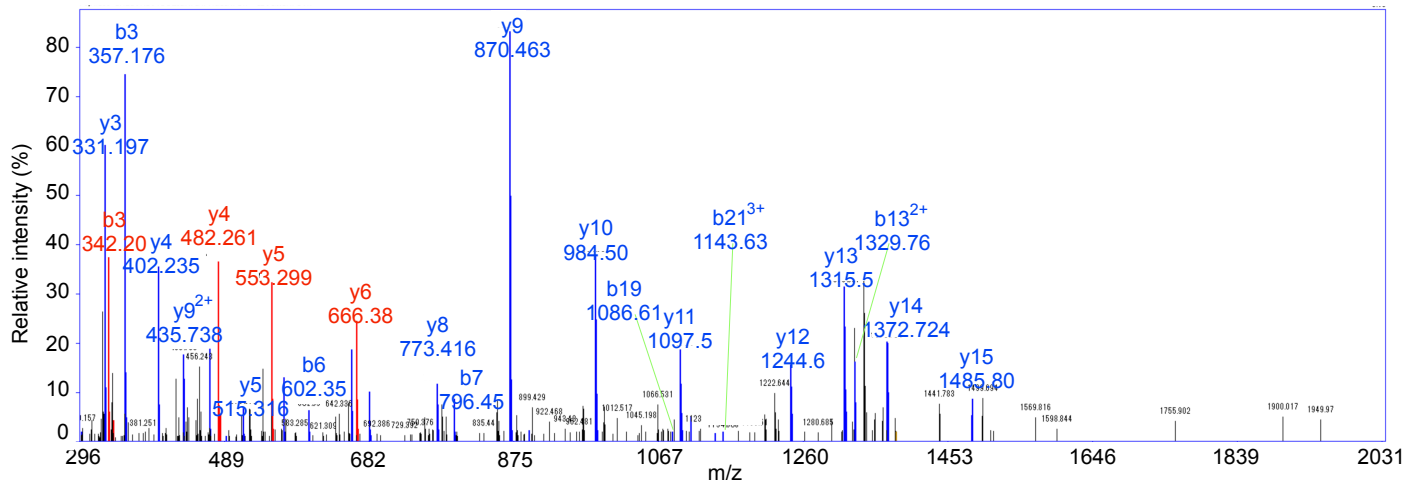

Supplement: S12 Fig — MS/MS spectrum of the peptide IEVGK(pBPa)LAFSTK (at positions 274 to 284 for native PamA) crosslinked to LNELVNLVTDLQIGAFINPNGSIAPS (at positions 247 to 273 for native PamA) in MBP-ΔN-PamA K278(pBPa). The crosslinked site was analyzed by SIM-XL (http://patternlabforproteomics.org/sim-xl). The sequence can be read from the annotated b and y ion series: b3, y3, y4, y5, y6 ions of the peptide IEVGK(pBPa)LAFSTK from MBP-ΔN-PamA K278(pBPa) (shown in red), and b3, b4, b5, b6, b7, b8, b10, b13, b19, b21, y4, y5, y6, y7, y8, y9, y10, y11, y12, y13, y14, y15 ions of a peptide LNELVNLVTDLQIGAFINPNGSIAPS from MBP-ΔN-PamA K278(pBPa) (blue) were observed, respectively. We could not confirm whether pBPa at position 278 was crosslinked to Leu at position 257 or Gln at position 258 (shown in yellow overlay). (PDF) [file pone.0237883.s012.pdf]

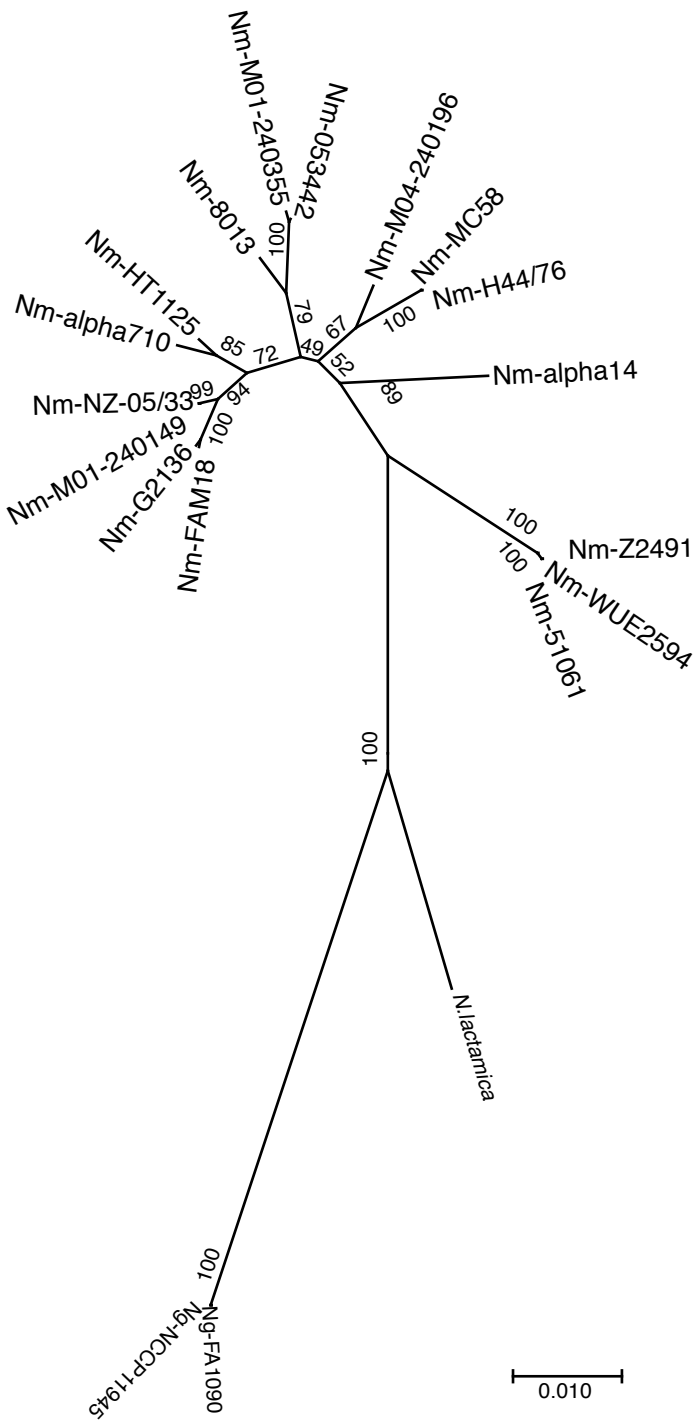

Supplement: S13 Fig — The pamA genes (the genes encoding “conserved hypothetical protein”) from three neisserial species were analyzed by Molecular Evolutionary Genetics Analysis (MEGA) X [92]. The evolutionary history was inferred using the Neighbor-Joining method [93]. The tree is drawn to scale, with branch lengths in the same units as those of the evolutionary distances used to infer the phylogenetic tree. The evolutionary distances were computed using the Maximum Composite Likelihood method [94] and are in the units of the number of base substitutions per site. The robustness of the NJ method was tested by bootstrapping with 500 replicates of data, and the percentages are shown at the nodes. This analysis involved 19 nucleotide sequences of genes encoding N. meningitidis HT1125 PamA (DDBJ accession no. LC511747), N. meningitidis H44/76 conserved hypothetical protein (GenBank accession no. CP002420), N. meningitidis MC58 hypothetical protein (GenBank accession no. AE002098), N. meningitidis M04-240196 conserved hypothetical protein (GenBank accession no. CP002423), N. meningitidis alpha14 conserved hypothetical protein (GenBank accession no. AM889136), N. meningitidis NZ-05/33 conserved hypothetical protein (GenBank accession no. CP002424), N. meningitidis M01-240149 conserved hypothetical protein (GenBank accession no. CP002421), N. meningitidis alpha710 hypothetical protein (GenBank accession no. CP001561), N. meningitidis 053442 conserved hypothetical protein (GenBank accession no. CP000381), N. meningitidis 8013 conserved hypothetical protein (GenBank accession no. FM999788), N. meningitidis M01-240355 conserved hypothetical protein (GenBank accession no. CP002422), N. meningitidis 510612 hypothetical protein (GenBank accession no. CP007524), N. meningitidis WUE2694 conserved hypothetical protein (GenBank accession no. FR774048), N. meningitidis G2136 conserved hypothetical protein (GenBank accession no. CP002419), N. meningitidis Z2491 hypothetical protein (GenBank accession no. [file pone.0237883.s013.pdf]
