# Supplementary material for: Genetic incorporation of non-canonical amino acid photocrosslinkers in Neisseria meningitidis: New method provides insights into the physiological function of the function-unknown NMB1345 protein
Source: PLoS One. 2020 Aug 31;15(8):e0237883. doi: 10.1371/journal.pone.0237883 (PMC7458321; doi:10.1371/journal.pone.0237883)
Supplement: S4 Fig — (A) Amino acid sequence of the C-terminal region of PamA K278-Strep2-His6. (B) Nucleotide sequence of the C-terminal region of PamA K278-Strep2-His6. The same colors in A and B corresponds to the same region in amino acid (A) and nucleotide (B) sequences. (PDF) [file pone.0237883.s004.pdf]

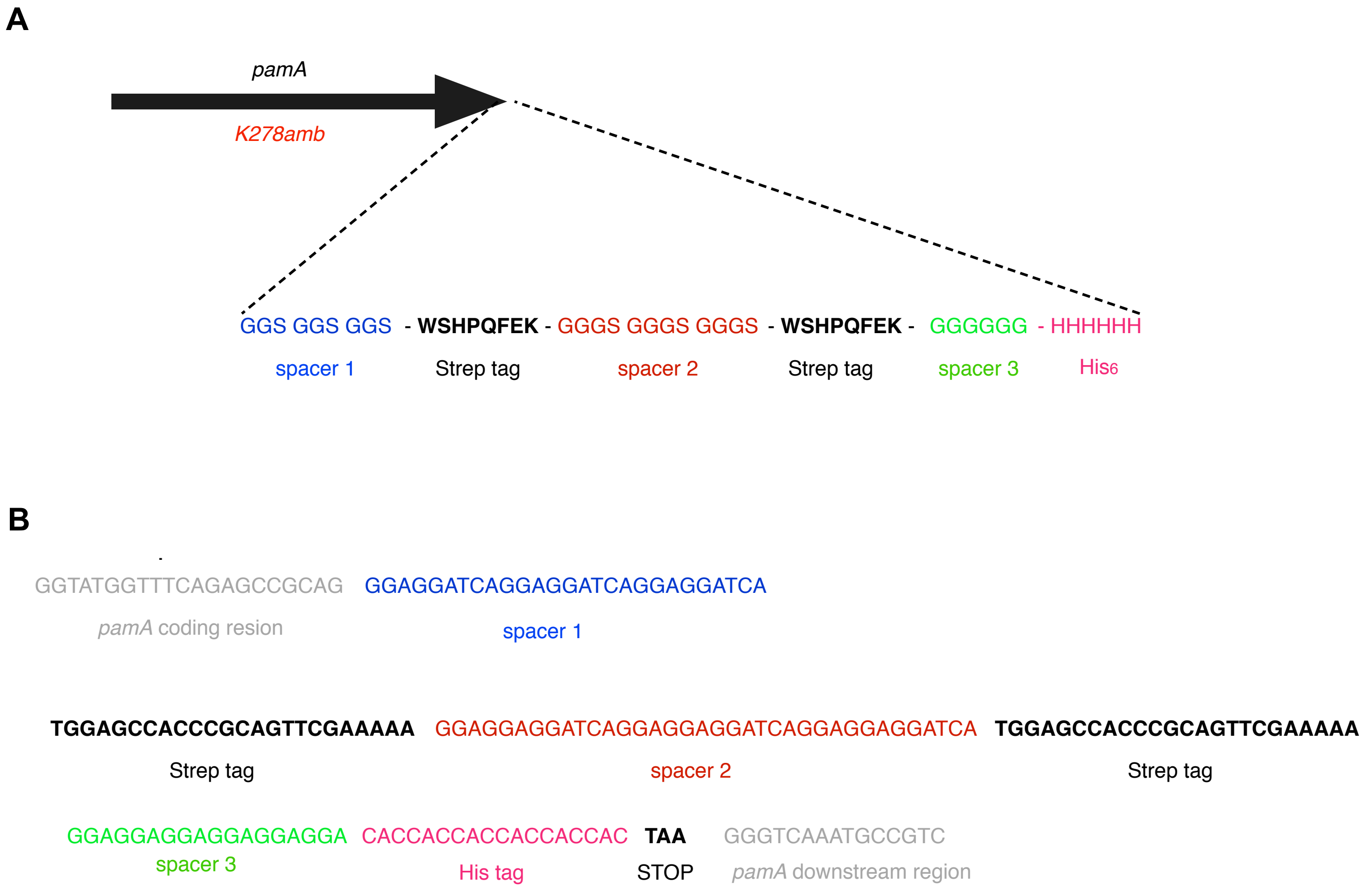

A
pamA
K278amb
GGS GGS GGS - WSHPQFEK - GGGS GGGS GGGS - WSHPQFEK - GGGGGG - HHHHHH
spacer 1
Strep tag
spacer 2
Strep tag
spacer 3
His6
B
GGTATGGTTTCAGAGCCGCAG
pamA coding resion
GGAGGATCAGGAGGATCAGGAGGATCA
spacer 1
TGGAGCCACCCGCAGTTCGAAAAA
Strep tag
GGAGGAGGATCAGGAGGAGGATCAGGAGGAGGATCA
spacer 2
TGGAGCCACCCGCAGTTCGAAAAA
Strep tag
GGAGGAGGAGGAGGAGGA
spacer 3
CACCACCACCACCACCAC
His tag
TAA
STOP
GGGTCAAATGCCGTC
pamA downstream region
