# Supplementary material for: Genetic incorporation of non-canonical amino acid photocrosslinkers in Neisseria meningitidis: New method provides insights into the physiological function of the function-unknown NMB1345 protein
Source: PLoS One. 2020 Aug 31;15(8):e0237883. doi: 10.1371/journal.pone.0237883 (PMC7458321; doi:10.1371/journal.pone.0237883)

A

1 MKKPLISVAAALLGVALGTPYYLGVKAEESLTQQQKILQEAGFLTVESHQ 50
51 YERGWFTSTETTVIRLKPELLNNARKYLPDNLKTVLEQPVTLVNHITHGP 100
101 FAGGFGTQAYIETEFKYAPETEKVLERFFGKQAPVSLANTVYFNGSGKME 150
151 VSVPAFDYEELSGIRLHWEGLTGKTVYQKGFKSYRNSYDAPLFKIKLADK 200
201 GDAAFEKAHFDSETSDGINPLALGSSNLTLEKFSLEWKEGVDYNVKLNEL 250
251 VNLVTDLQIGAFINPNGSIAPSKIEVGXLAFSTKTGESGSFINSEGQFRF 300
301 DTLVYGNEKYGPLDIHIAAEHLDASALTVLKRKFAQISAKKMTEEQIRND 350
351 LIAAVKGEASGLFTNNPVLDIKTFRFTQPSGKIDVGGKIMFKDMKKEDLN 400
401 QLGLMLKKTEADIRMSIPQKMLEDLAVSQAGNIFSVNAEDEAEGRASLDD 450
451 INETLRLMVDSTVQSMAREKYLTLNGDQIDTAISLKNNQLKLNGKTLQNE 500
501 PEPDFDEGGMVSEPQQGGSGGSGGSWSHPQFEKGGGSGGGSGGGSWSHPQ 550
551 FEKGGGGGGHHHHHH 564

B

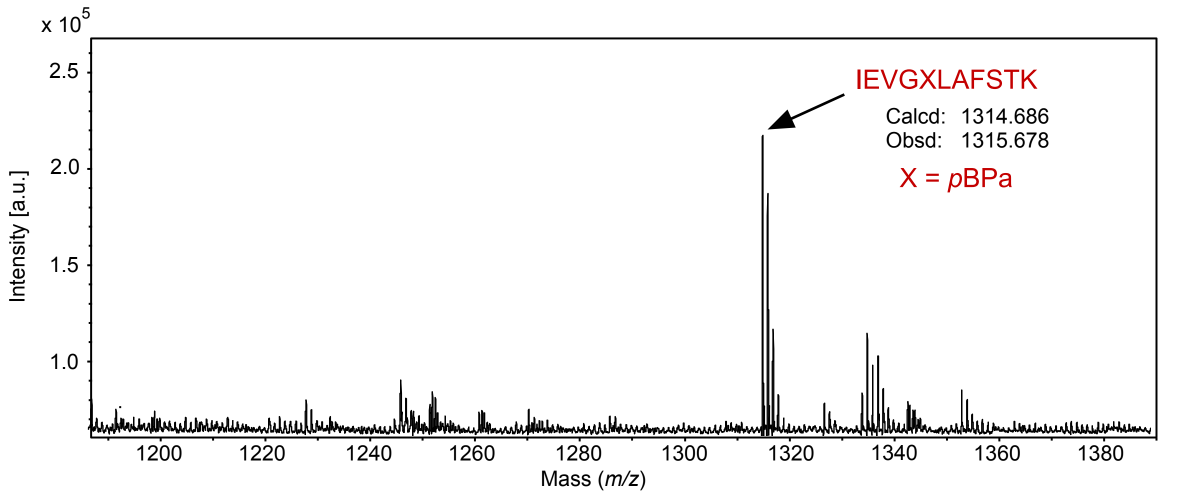

C

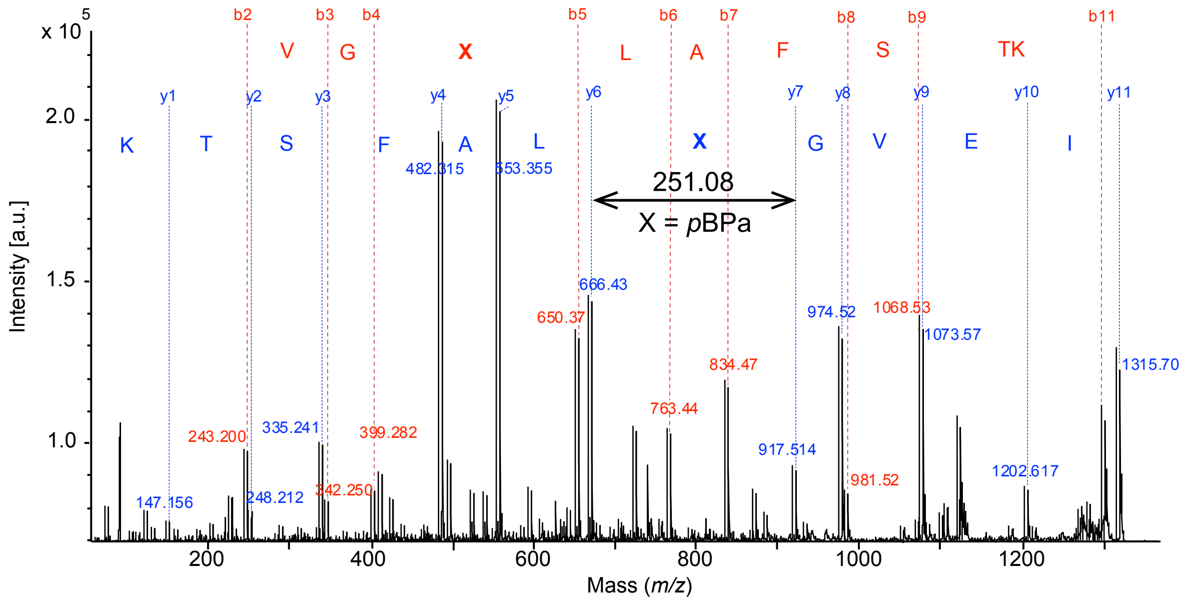

Supplement: S5 Fig — (A) Amino acid sequence of PamA with Strep2-His6 tag at C-terminus. The tryptic peptide containing a ncAA, pBPa is highlighted in red and the pBPa at position 278 is represented as X. (B) The incorporation of pBPa at position 278 was confirmed by MALDI-TOF MS analysis of the tryptic peptide IEVGXLAFSTK (X represents pBPa). The observed (obsd) molecular masses agreed well with the calculated (calcd) masses. (C) MALDI-TOF MS/MS analysis of the tryptic peptide of shown in B. Tandem mass spectrum of the peptide IEVGXLAFSTK (X = pBPa). The sequence can be read from the annotated b (red) or y (blue) ion series. (PDF) [file pone.0237883.s005.pdf]
