# Supplementary material for: Genetic incorporation of non-canonical amino acid photocrosslinkers in Neisseria meningitidis: New method provides insights into the physiological function of the function-unknown NMB1345 protein
Source: PLoS One. 2020 Aug 31;15(8):e0237883. doi: 10.1371/journal.pone.0237883 (PMC7458321; doi:10.1371/journal.pone.0237883)
Supplement: S1 Table — (DOCX) [file pone.0237883.s014.docx]

**S1 Table Oligonucleotides used in this study**

**Cloning of *pamA^+^* and *ΔpamA* and ectopic complementation of *pamA^+^ at ggt* locus**

Primer target gene (vector) Nucleotide sequence (5′-3′) Reference

NMB1345-21 *pamA* TCGGATGGGTACGGTTTACAGCAC This study

NMB1345-2 *pamA* CCCGGGCTACTGGCGCGGTCA This study

NMB1345(pET303)-1(NsiI)# *pamA* CAAATGCATGAAGAAAGCTTGACGCAGCAG This study

NMB1345(pET303)-2(XhoI)# *pamA* CGGCTCGAGCTGCTGCGGCTCTGAAACCAT This study

NMB1345-3 *pamA* ACTTCAAACAAGCAGGTTTCAAAGACGCT This study

NMB1345-4 *pamA* TGGGTCAAATGCCGTCTGAAATGTTCAGGC This study

M13-RV-ggt-5′ *ggt* ATTTTCACACAACCCAGGAAACAGGATGACCATGAT This study

M13-47-ggt-3′ *ggt* ATCATCCCCACCCCGGCCGAGGGTTTTCCCAGTCAC This study

ggt-3 *ggt* GACTGCTGATGACATTAGCGG [45]

ggt-4 *ggt* GATTACTCACAATTTCCCCCTA [45]

**Construction for *pilE^+^-cat pilE I12A, pilE^+^-ermC and pilE-I12A-ermC***

Primer target gene (vector) Nucleotide sequence (5′-3′) Reference

pGEM-3z-(SmaI)-2(15mer)-pilE-51 *pilE* TCGAGCTCGGTACCCTCCGACCCAATCAACACACCCGATGCC This study

pilE-52 *pilE* TTTGCCTTAGGCAGTAGCACTTGT This study

piE-53 *pilE* TTATACCATAAATTTTAAATAAATCAAGCG This study

ermC-21 *ermC* ACTGCCTAAGGCAAAAAGAGGGTTATAATGAACGAGAAAAAT This study

ermC-22 *ermC* AAATTTATGGTATAATTACTTATTAAATAATTTATAGCTATT This study

pilE-18 *pilE* CTCTAGAGGATCCCCCGGACGACCCCGCTTGCCTTG [36]

pilE I12A-1 *pilE* ATTGCC**GC**CGTCGGCATTTTGGCGGCAGTC This study

pilE I12A-2 *pilE* GCCGACG**GC**GGCAATCACAATCATCAGCTC This study

T7 pGEM-3z TAATACGACTCACTATAGGG Promega

SP6 pGEM-3z CAAGCTATTTAGGTGACACTATAG Promega

***pamA’-lacZ***

Primer target gene (vector) Nucleotide sequence (5′-3′) Reference

lacZ-21 *lacZ* ATGACCATGATTACGGATTCACTGGCC This study

M13-RV’(15mer)-lacZ-22 *lacZ* GGGAAAACCCTGGCGGGTCAAATGCCGTCTGAAATG This study

M13-47 *lacZ* CGCCAGGGTTTTCCCAGTCACGAC Takara Bio

M13-RV *lacZ* CAGGAAACAGCTATGA TakaraBio

M13-47′(15mer)-nmb1345-61 *pamA* GGGAAAACCCTGGCGGGTCAAATGCCGTCTGAAATG This study

lacZ′-21(15mer)-nmb1345-62 *pamA* CGTAATCATGGTCATGCGCTCATATTGGTGCGATTC This study

**Constriction for *pamA’-phoA***

Primer target gene (vector) Nucleotide sequence (5′-3′) Reference

phoA-21 *phoA* GCTCAGGGCGATATTACTGCA This study

M13-RV′(15mer)-phoA-2 *phoA* CATAGCTGTTTCCTGTTATTTCAGCCCCAGAGCGGCTTT This study

nmb1345-5 *pamA* ATGGTGGTTAAGGGGGTACAAGTC This study

nmb1345-21 *pamA* CGGTTTACAGCACGCTGGGTT This study

**IncQ plasmid containing *lacI^q^-P_tac_-pylRS[Y306A/Y384F]/T***

Primer target gene (vector) Nucleotide sequence (5′-3′) Reference

ptac-12-pylRS-1 *pyrRS+T* ACACAGGAAACAGCGCATATGGATAAAAAACCACTAAAC This study

M13-47reverse-lppT-2 *pyrRS+T* GGTTTTCCCAGTCACGCCTTCTTGACGAGTTCTTCTGAA This study

M13-47 reverse *lacZ* GTGACTGGGAAAACCCTGGCGT This study

ptac-12 pTTQ18 CGCTGTTTCCTGTGTGAAATTGTT This study

**IncQ plasmid containing *lacI^q^-P_tac_-MmpBPaRS*/*T***

Primer target gene (vector) Nucleotide sequence (5′-3′) Reference

A302T-F *pylRS* CTGAGACCCATGCTT**A**CTCCAAACCTTTACAACTACCTGCGCA This study

A302T-R *pylRS* GTTGTAAAGGTTTGGAG**T**AAGCATGGGTCTCAGGCAGAAGTTC This study

N346T-C348T-F *pylRS* TCGAAGAGTTTACCATGCTGA**C**CTTC**AC**CCAGATGG

　　　　　　　　　　　　　　　 GATCGGGATGCACACGGGAAAAT This study

N346T-C348T-R *pylRS* GTGCATCCCGATCCCATCTGG**GT**GAAG**G**TCAGCATGGTAAACT

CTTCGAGGTGTTCTTT

W417C-F *pylRS* GGTATTGATAAACCCTG**T**ATAGGGGCAGGTTTCGGGCTCGAAC This study

W417C-R *pylRS* GAAACCTGCCCCTAT**A**CAGGGTTTATCAATACCCCATTCCCGG This study

***gst* amber mutants**

Primer target gene (vector) Nucleotide sequence (5′-3′) Reference

gst E51amb-1 *gst* GGGTTTTG**T**AGTTTCCCAATCTTCCTT This study

gst E51amb-2 *gst* GGAAACT**A**CAAACCCAATTCAAACT This study

gst F52amb-1 *gst* TTGGAGT**AG**CCCAATCTTCCTTATTATAT This study

gst F52amb-2 *gst* AGATTGGG**CT**ACTCCAAACCCAATTCAA This study

tetA-1-gst-up pGEX-6P-1 AAGGGAGAGCGTCGAAATTCGTGTCGCTCAAGCGCCAC This study

gst-down-tetA-2 pGEX-6P-1 AAGGGCATCGGTCGATCAAGAATTATACATTCCGCTATC This study

tetA-1-ptac-13 pTTQ18 **AAGGGAGAGCGTCGACCCCGCGCGTTGGCCGATTCATTA** This study

tetA-2-ptac-14  pTTQ18 **AAGGGCATCGGTCGACGGCATCAGAGCAGATTGTACTGA** This study

***pamA K-amb* mutant series**

Primer target gene (vector) Nucleotide sequence (5′-3′) Reference

NMB1345-1 *pamA* TGGGTCAAATGCCGTCTGAAATGT This study

NMB1345-2(His)_6_ *pamA* **TTAGTGGTGGTGGTGGTGGTGCTGCTGCGGCTCTGAAACCATAC** This study

nmb1345 K148 amber -1 *pamA* AGCGGT**TAG**ATGGAAGTCAGTGTTCCC This study

nmb1345 K148 amber -2 *pamA* TTCCAT**CTA**ACCGCTGCCGTTAAAATA This study

nmb1345 K273 amber -1 *pamA* CCTTCC**TAG**ATCGAAGTCGGCAAA This study

nmb1345 K273 amber -2 *pamA* TTCGAT**CTA**GGAAGGTGCGATGCT This study

nmb1345 K388 amber -1 *pamA* GGCGGA**TAG**ATCATGTTTAAAGACATGAAG This study

nmb1345 K388 amber -2 *pamA* CATGAT**CTA**TCCGCCCACATCGATTTTTCC This study

nmb1345 K3 amber -1 *pamA* ATGAAA**TAG**CCTTTGATTTCG This study

nmb1345 K3 amber -2 *pamA* CAAAGG**CTA**TTTCATCACTTC This study

nmb1345 K174 amber -1 *pamA* ACGGGA**TAG**ACGGTTTATCAA This study

nmb1345 K174 amber -2 *pamA* AACCGT**CTA**TCCCGTCAATCC This study

nmb1345 K179 amber -1 *pamA* TATCAA**TAG**GGTTTCAAAAGC This study

nmb1345 K179 amber -2 *pamA* GAAACC**CTA**TTGATAAACCGT This study

nmb1345 K182 amber -1 *pamA* GGTTTC**TAG**AGCTACCGCAAC This study

nmb1345 K182 amber -2 *pamA* GTAGCT**CTA**GAAACCTTTTTG This study

nmb1345 K194 amber -1 *pamA* TTGTTC**TAG**ATCAAGCTGGCA This study

nmb1345 K194 amber -2 *pamA* GCTTGA**CTA**TGAACAAGGGCG This study

nmb1345 K208 amber -1 *pamA* TTTGAA**TAG**GCGCATTTCGAT This study

nmb1345 K208 amber -2 *pamA* ATGCGC**CTA**TTCAAACGCGGC This study

nmb1345 K232 amber -1 *pamA* TTGGAA**TAG**TTCTCCCTAGAA This study

nmb1345 K232 amber -2 *pamA* GGAGAA**CTA**TTCCAAGGTCAG This study

nmb1345 K238 amber -1 *pamA* GAATGG**TAG**GAGGGTGTCGAT This study

nmb1345 K238 amber -2 *pamA* ACCCTC**CTA**CCATTCTAGGGA This study

nmb1345 K246 amber -1 *pamA* AACGTC**TAG**TTAAACGAACTG This study

nmb1345 K246 amber -2 *pamA* GTTTAA**CTA**GACGTTGTAATC This study

nmb1345 K278 amber -1 *pamA* GTCGGC**TAG**CTGGCTTTTTCA This study

nmb1345 K278 amber -2 *pamA* AGCCAG**CTA**GCCGACTTCGAT This study

nmb1345 K284 amber -1 *pamA* TCAACC**TAG**ACCGGGGAATCA This study

nmb1345 K284 amber -2 *pamA* CCCGGT**CTA**GGTTGAAAAAGC This study

nmb1345 K309 amber -1 *pamA* AATGAA**TAG**TACGGCCCGCTG This study

nmb1345 K309 amber -2 *pamA* GCCGTA**CTA**TTCATTGCCGTA This study

nmb1345 K331 amber -1 *pamA* GTATTG**TAG**CGTAAGTTTGCA This study

nmb1345 K331 amber -2 *pamA* CTTACG**CTA**CAATACGGTTAA This study

nmb1345 K341 amber -1 *pamA* GCCAAA**TAG**ATGACCGAGGAA This study

nmb1345 K341 amber -2 *pamA* GGTCAT**CTA**TTTGGCGGAAAT This study

nmb1345 K356 amber -1 *pamA* GCCGTC**TAG**GGCGAGGCTTCC This study

nmb1345 K356 amber -2 *pamA* CTCGCC**CTA**GACGGCGGCAAT This study

nmb1345 K371 amber -1 *pamA* GACATT**TAG**ACTTTTCGTTTC This study

nmb1345 K371 amber -2 *pamA* AAAAGT**CTA**AATGTCCAATAC This study

nmb1345 K382 amber -1 *pamA* TCGGGA**TAG**ATCGATGTGGGT This study

nmb1345 K382 amber -2 *pamA* ATCGAT**CTA**TCCCGACGGCTG This study

nmb1345 K395 amber -1 *pamA* GACATG**TAG**AAGGAAGATTTG This study

nmb1345 K395 amber -2 *pamA* TTCCTT**CTA**CATGTCTTTAAA This study

nmb1345 K396 amber -1 *pamA* ATGAAG**TAG**GAAGATTTGAAT This study

nmb1345 K396 amber -2 *pamA* ATCTTC**CTA**CTTCATGTCTTT This study

nmb1345 K407 amber -1 *pamA* ATGCTG**TAG**AAAACCGAAGCC This study

nmb1345 K407 amber -2 *pamA* GGTTTT**CTA**CAGCATCAAACC This study

nmb1345 K408 amber -1 *pamA* CTGAAG**TAG**ACCGAAGCCGAC This study

nmb1345 K408 amber -2 *pamA* TTCGGT**CTA**CTTCAGCATCAA This study

nmb1345 K420 amber -1 *pamA* CCCCAA**TAG**ATGCTGGAAGAC This study

nmb1345 K420 amber -2 *pamA* CAGCAT**CTA**TTGGGGAATACT This study

nmb1345 K470 amber -1 *pamA* AGGGAA**TAG**TATCTGACTTTG This study

nmb1345 K470 amber -2 *pamA* CAGATA**CTA**TTCCCTTGCCAT This study

nmb1345 K486 amber -1 *pamA* TCTCTG**TAG**AACAATCAGTTG This study

nmb1345 K486 amber -2 *pamA* ATTGTT**CTA**CAGAGAAATGGC This study

nmb1345 K491 amber -1 *pamA* CAGTTG**TAG**TTGAACGGTAAA This study

nmb1345 K491 amber -2 *pamA* GTTCAA**CTA**CAACTGATTGTT This study

nmb1345 K495 amber -1 *pamA* AACGGT**TAG**ACGTTGCAAAAC This study

nmb1345 K495 amber -2 *pamA* CAACGT**CTA**ACCGTTCAATTT This study

tetA-NMB1345-up *pamA* AAGGGAGAGCGTCGAGGCGTGGATGGGATACCCCGTTTG  This study

tetA-2-NMB1345-down  *pamA* **AAGGGCATCGGTCGATTGGGCGAATCCATCGGTATGGCG**  This study

spacer-1(15mer)-nmb1345-12 *pamA* TCCTCCTGATCCTCCCTGCGGCTCTGAAACCATACC This study

His_6_′(15mer)-nmb1345-11 *pamA* CACCACCACCACTAATGGGTCAAATGCCGTCTGAAA This study

***pilE-FLAG, pilF-FLAG, pilF-FLAG, pilM-HA and pilX-FLAG* mutants’ series**

Primer target gene (vector) Nucleotide sequence (5′-3′) Reference

pilE-11 *pilE* AACCCACCCTATGCTACCGCGCAAATTCAA This study

pilE-12 *pilE* TCATTCCCACAAGGACAAAAAACCAAAACA This study

FLAG-RV-M pUC18(ermC) **GACTACAAAGACGATGACGACAAGTAA**

**GAGCGGATAACAATTTCACACAGG** This study

pilE-17'(15mer)-M13-47 pUC18(ermC) ATGGTATAATTTGCCCGCCAGGGTTTTCCCAGTCACGAC This study

pilE-17 *pilE* GGCAAATTATACCATAAATTTTAAATA This study

FLAG′(15mer)-pilE-18 *pilE* ATCGTCTTTGTAGTCGGCAGTAGCACTTGTATCGCG This study

M13-RV'(15mer)-pilE-18 *pilE* CATAGCTGTTTCCTGAAAATTACTCCTAATTGGAAA This study

M13-47'(15mer)-pilE-19 *pilE*  GGGAAAACCCTGGCGGGCAAATTATACCATAAATTT This study

pMW(SmaI)-up(15mer)-pilF-1 *pilF* TCGAGCTCGGTACCCGACAGGTTGCGCGATGTCATGCCG This study

pMW(SmaI)-down(15mer)-pilF-2 *pilF* CTCTAGAGGATCCCCTCCCAATAGAACGGCTTTTTCGGC This study

pilF-3 *pilF* ACAGATTCAAACCATATGAAC This study

FLAG′(15mer)-pilF-4 *pilF* ATCGTCTTTGTAGTCTTGACCGGTGAGGACGGTTTG This study

pilF-3′(15mer)-M13-47 pUC18(ermC) ATGGTTTGAATCTGTCGCCAGGGTTTTCCCAGTCACGAC This study

pMW(SmaI)-up(15mer)-pilMNOP-3 *pilMNOP* TCGAGCTCGGTACCCCGCCGGCGAAGCTGTGTTGGCCGT This study

pMW(SmaI)-down(15mer)-pilMNOP-4 *pilMNOP* CTCTAGAGGATCCCCCCGCCGCCTGTTGTTTTGCCGGGC This study

pilP-3 *pilP* GAAGAGGATTACTCCATTATG This study

FLAG′(15mer)-pilP-2 *pilP* ATCGTCTTTGTAGTCATTTTGTTCTGCGGCAGGTGC This study

pilP-3′(15mer)-M13-47 *pilP* GGAGTAATCCTCTTCCGCCAGGGTTTTCCCAGTCACGAC This study

pilO-3 *pilO* CTTACGTTAGGGAAACCATGAAAC This study

HA′-pilM-2 *pilM* TTAAGCGTAATCCGGAACATCGTATGGGT

ATAATCCCGTACCGCCAAACC This study

HA′(15mer)-pilN-1 *pilN* CCGGTATACGCTTAATTATGAACAATTTAATCAAAATCA This study

pilO-2 *pilO* TTATTTTTGCTCGGCATTTTGTGC This study

pilO-2′(15mer)-M13-RV pUC18(ermC) GCCGAGCAAAAATAACGCCAGGGTTTTCCCAGTCACGAC This study

pilO-3′(15mer)-M13-47 pUC18(ermC) TTTCCCTAACGTAAGCAGGAAACAGCTATGA This study

pMW(SmaI)-up(15mer)-pilX-3 *pilX* TCGAGCTCGGTACCCGCCATACCTTCTTATCAAAGTTATAT This study

pMW(SmaI)-down(15mer)-pilX-6 *pilX* CTCTAGAGGATCCCCTGCTGAAGCACCAAGTGAATCGGTTCC This study

M13-47′(15mer)-pilX-5 *pilX* GGGAAAACCCTGGCGGGTTGTTTTGCCAATACCGTCTGA This study

FLAG′(15mer)-pilX-8 *pilX* ATCGTCTTTGTAGTCTTTTTTACGATTAGAGAAGGCTTC This study

***ΔN-pamA-K278amb***

Primer target gene (vector) Nucleotide sequence (5′-3′) Reference

NMB1345(pGEX)-1(BH) *pamA* CGGGATCCGAAGAAAGCTTGACGCAGCAG This study

NMB1345(pGEX)-2(XhoI) *pamA* GCCTCGAGTTACTGCTGCGGCTCTGAAAC This study

(Gln)_6_-pilE-45 *pilE* CAACAACAACAACAACAATTTACCCTTATGAGCTGATGATTGTG This study

pCDF-1b(PacI)(15mer)-pilE-44) pCDF-1b GCCTAGGTTAATTAATTAGCTGGCATCACTTGCGTCGCG This study

pCDF-1b (downstream of PacI) pCDF-1b TTAATTAACCTAGGCTGCTGCCACCGC This study

(Gln)_6_(15mer)-pCDF-1b-2 pCDF-1b TTGTTGTTGTTGTTGCATGGTATATCTCCTTATTAAAGTTA This study

pSTV28(SmaI-5′)-pCDF-1b-1 pCDF-1b TCGAGCTCGGTACCCGCTCTCCCTTATGCGACTCC This study

pSTV28(SmaI-3′)-pCDF-1b-2 pCDF-1b CTCTAGAGGATCCCCAAGTGCCACTTGCGGAGACCCGGT This study

*The nucleotides shown in underlined indicate 15 mer nucleotides originally added for InFusion Cloning.

** The nucleotides shown in double-underlined letter indicate His_6_, FLAG or HA Tag sequence, respectively.

# The nucleotides shown in bold underlined indicate the restriction enzyme sites.

## The nucleotides shown in bold case indicate the introduced mutations by site-directed mutagenesis.
