# Supplementary material for: Genetic incorporation of non-canonical amino acid photocrosslinkers in Neisseria meningitidis: New method provides insights into the physiological function of the function-unknown NMB1345 protein
Source: PLoS One. 2020 Aug 31;15(8):e0237883. doi: 10.1371/journal.pone.0237883 (PMC7458321; doi:10.1371/journal.pone.0237883)
Supplement: S2 Table — (DOCX) [file pone.0237883.s015.docx]

| **S2 Table Plasmids to construct *N. meningitidis* mutants** | |  |  |  |
| --- | --- | --- | --- | --- |
| Plasmid | Relative properties | Antibiotic selection marker | References |  |
| pTWV228 | Cloning vector | Amp | Takara Bio |  |
| pHT922 | Derivative of pTWV228 carrying a *pamA* gene with the flanking region | Amp | This study |  |
| pHT930 | Derivative of pHT922 carrying *ΔpamA::spc* | Amp | This study |  |
| pHT195 | Derivative of pTWV228 carrying a *ggt* gene | Amp | This study |  |
| pHT923 | Derivative of pHT195 carrying *pamA^+^- cat* gene a | Amp | This study |  |
| pHT924 | Derivative of pHT195 carrying *ggt::pamA^+^- cat* genes at BstXI sites | Amp | This study |  |
| pHT24 | Derivative of pUC18 carrying *ermC gene* | Amp, Erm | This study |  |
| pHT1605 | Derivative of pTWV228 carrying *pamA '-lacZ-ermC* genes | Amp, Erm | This study |  |
| pHT1603 | Derivative of pTWV228 carrying *pamA '-phoA-ermC* genes | Amp, Erm | This study |  |
| pTTQ18 | Expression vector | Amp | [51] |  |
| pHT1208 | Derivative of pTTQ18 carrying *lacI^q^-P_tac_-pylRS[Y306A/384F]/T* | Amp | This study |  |
| pHT1261 | Derivative of pTTQ18 carrying *lacI^q^-P_tac_-MmpBPaRS*/*T* | Amp | This study |  |
| pGEM-T-3z | Cloning vector | Amp | Promega |  |
| pHT872 | Derivative of pGEM-T-3z carrying a *pilE^+-^cat* translational gene | Amp Cml | [36] |  |
| pHT1536 | Derivative of pHT872 carrying *pilE I12A-cat* translational genes | Amp, Cml | This study |  |
| pHT1504 | Derivative of pGEM-T-3z carrying a *pilE^+-^ermC* translational gene | Amp, Erm | This study |  |
| pHT1536 | Derivative of pHT872 carrying *pilE I12A-ermC-*translational genes | Amp, Erm | This study |  |
| pGEX-6P-1 | Expression vector for GST fusion | Amp | GE Healthcare |  |
| pHT1353 | Derivative of pGEX-6P-1carrying *gstE51amb* | Amp | This study |  |
| pHT1354 | Derivative of pGEX-6P-1carrying *gstF52amb* | Amp | This study |  |
| pHT1363 | Derivative of pTTQ18 carrying meningococcal *opaD^+^* gene | Amp | This study |  |
| pHT1364 | Derivative of pTTQ18 carrying meningococcal *opaD K95amb* | Amp | This study |  |
| pHT1213 | Derivative of pHT922 carrying a *pamA^+^-His_6_* gene | Amp | This study |  |
| pHT1234 | Derivative of pHT922 carrying a *pamA K3amb-His_6_* gene | Amp | This study |  |
| pHT1217 | Derivative of pHT922 carrying a *pamA K148amb-His_6_* gene | Amp | This study |  |
| pHT1235 | Derivative of pHT922 carrying a *pamA* *K174amb-His_6_* gene | Amp | This study |  |
| pHT1236 | Derivative of pHT922 carrying a *pamA K179amb-His_6_* gene | Amp | This study |  |
| pHT1237 | Derivative of pHT922 carrying a *pamA K182amb-His_6_* gene | Amp | This study |  |
| pHT1238 | Derivative of pHT922 carrying a *pamA K194amb-His_6_* gene | Amp | This study |  |
| pHT1239 | Derivative of pHT922 carrying a *pamA K208amb-His_6_* gene | Amp | This study |  |
| pHT1240 | Derivative of pHT922 carrying a *pamA K232amb-His_6_* gene | Amp | This study |  |
| pHT1241 | Derivative of pHT922 carrying a *pamA K246amb-His_6_* gene | Amp | This study |  |
| pHT1218 | Derivative of pHT922 carrying a *pamA K273amb-His_6_* gene | Amp | This study |  |
| pHT1242 | Derivative of pHT922 carrying a *pamA K278amb-His_6_* gene | Amp | This study |  |
| pHT1243 | Derivative of pHT922 carrying a *pamA K284amb-His_6_* gene | Amp | This study |  |
| pHT1244 | Derivative of pHT922 carrying a *pamA K309amb-His_6_* gene | Amp | This study |  |
| pHT1245 | Derivative of pHT922 carrying a *pamA K331amb-His_6_* gene | Amp | This study |  |
| pHT1247 | Derivative of pHT922 carrying a *pamA K341amb-His_6_* gene | Amp | This study |  |
| pHT1248 | Derivative of pHT922 carrying a *pamA K356amb-His_6_* gene | Amp | This study |  |
| pHT1249 | Derivative of pHT922 carrying a *pamA K371amb-His_6_* gene | Amp | This study |  |
| pHT1250 | Derivative of pHT922 carrying a *pamA K382amb-His_6_* gene | Amp | This study |  |
| pHT1252 | Derivative of pHT922 carrying a *pamA K395amb-His_6_* gene | Amp | This study |  |
| pHT1254 | Derivative of pHT922 carrying a *pamA K407amb-His_6_* gene | Amp | This study |  |
| pHT1255 | Derivative of pHT922 carrying a *pamA* *K408amb-His_6_* gene | Amp | This study |  |
| pHT1256 | Derivative of pHT922 carrying a *pamA K420amb-His_6_* gene | Amp | This study |  |
| pHT1257 | Derivative of pHT922 carrying a *pamA K470amb-His_6_* gene | Amp | This study |  |
| pHT1258 | Derivative of pHT922 carrying a *pamA K486amb-His_6_* gene | Amp | This study |  |
| pHT1259 | Derivative of pHT922 carrying a *pamA K491amb-His_6_* gene | Amp | This study |  |
| pHT1260 | Derivative of pHT922 carrying a *pamA K495amb-His_6_* gene | Amp | This study |  |
| pHT1219 | Derivative of pHT922 carrying a *pamA K388amb-His_6_* gene | Amp | This study |  |
| pHT1293 | Derivative of *pamA K278amb-StrepTag_2_-His_6_* gene | Amp | This study |  |
| pMW119 | Cloning vector | Amp | Nippon gene |  |
| pHT1419 | Derivative of pMW119 carrying a *pilE^+^* gene with the flanking region | Amp | This study |  |
| pHT1420 | Derivative of pMW119 carrying *pilE^+-^FLAG-ermC* | Amp, Erm | This study |  |
| pHT1499 | Derivative of pMW119 carrying *pilF^+^* allele | Amp | This study |  |
| pHT1457 | Derivative of pMW119 carrying *pilF^+^-FLAG-ermC* | Amp, Erm | This study |  |
| pHT1450 | Derivative of pMW119 carrying partial *pilM, pilN^+^, pilO^+^, pilP^+^* allele | Amp | This study |  |
| pHT1454 | Derivative of pMW119 carrying *pilP^+^-FLAG-ermC* | Amp, Erm | This study |  |
| pHT1453 | Derivative of pMW119 carrying partial *pilM-HA* p*ilM^,^ pilN^+^, pilO^+-^ermC, pilP^+^* allele | Amp, Erm | This study |  |
| pHT1594 | Derivative of pMW119 carrying partial *pilX* gene | Amp | This study |  |
| pHT1597 | Derivative of pMW119 carrying *pilX^+^-FLAG -ermC* genes | Amp, Erm | This study |  |

Amp and Erm stand for ampicillin and erythromycin resistance marker, respectively.
