# Supplementary material for: Genetic incorporation of non-canonical amino acid photocrosslinkers in Neisseria meningitidis: New method provides insights into the physiological function of the function-unknown NMB1345 protein
Source: PLoS One. 2020 Aug 31;15(8):e0237883. doi: 10.1371/journal.pone.0237883 (PMC7458321; doi:10.1371/journal.pone.0237883)
Supplement: S3 Table — (DOCX) [file pone.0237883.s016.docx]

| **S3 Table Plasmids for production of protein recombinants expressed in *E. coli*** | |  |  |
| --- | --- | --- | --- |
|  |  |  |  |
|  | |  |  |
| Plasmid | Relative properties | Antibiotic selection marker | References |
| pET303CT-His | Expression vector | Amp | Invitrogen |
| pHT934 | Derivative of pET303CT-His expressing a *pamA* gene deleted 69 bp at 5´terminus (ΔN-PamA) | Amp | This study |
| pMAL-c2 | Expression vector for MBP fusion | Amp | NEB |
| pHT1473 | Derivative of pMAL-c2 expressing a MBP- ΔN-PamA K278amb | Amp | This study |
| pCDF-1b-kan | Expression vector | Kan | [53] |
| pCDF-Pyl-Fx3 | Derivative of pCDF-1b-kan expressing *P_glnS_-MmPylRS([A302T/N346T/C348T/W417C])* and two *P_lpp_-tRNA^pyl^* genes | Kan | This study |
| pSTV29 | Cloning vector | Cml | Takara Bio |
| pHT1447 | Derivative of pCDF-1b carrying *P_T7_- ΔN-(Gln)_6_-pilE* gene | Kan | This study |
| pHT1474 | Derivative of pSTV29 expressing *pilE* gene replaced the first 27 bp with six CAA repeats (6 Gln ) under the control of T7 promoter | Cml | This study |

Amp, Kan and Cml stand for ampicillin, kanamycin and chloramphenicol resistance marker, respectively.
