# Supplementary material for: Genetic incorporation of non-canonical amino acid photocrosslinkers in Neisseria meningitidis: New method provides insights into the physiological function of the function-unknown NMB1345 protein
Source: PLoS One. 2020 Aug 31;15(8):e0237883. doi: 10.1371/journal.pone.0237883 (PMC7458321; doi:10.1371/journal.pone.0237883)
Supplement: S4 Table — (DOCX) [file pone.0237883.s017.docx]

| **S4 Table IncQ plasmids for *N. meningitidis* strains which results were shown in supporting information** | | | |
| --- | --- | --- | --- |
| Plasmid | Relative properties | Antibiotic selection marker | References |
| pHT1216 | Derivative of pHT1212 carrying a *pamA^+^-His_6_* gene | Cml | This study |
| pHT1220 | Derivative of pHT1212 carrying a *pamA K148amb-His_6_* gene | Cml | This study |
| pHT1221 | Derivative of pHT1212 carrying a *pamA K273amb-His_6_* gene | Cml | This study |
| pHT1222 | Derivative of pHT1212 carrying a pamA *K388amb-His_6_* gene | Cml | This study |
| pHT1263 | Derivative of pHT1262 carrying a *pamA^+^-His_6_* gene | Cml | This study |
| pHT1264 | Derivative of pHT1262 carrying a *pamA K3amb-His_6_* gene | Cml | This study |
| pHT1265 | Derivative of pHT1262 carrying a *pamA K148amb-His_6_* gene | Cml | This study |
| pHT1266 | Derivative of pHT1262 carrying a *pamA* *K174amb-His_6_* gene | Cml | This study |
| pHT1267 | Derivative of pHT1262 carrying a *pamA K179amb-His_6_* gene | Cml | This study |
| pHT1268 | Derivative of pHT1262 carrying a *pamA K182amb-His_6_* gene | Cml | This study |
| pHT1269 | Derivative of pHT1262 carrying a *pamA K194amb-His_6_* gene | Cml | This study |
| pHT1271 | Derivative of pHT1262 carrying a *pamA K232amb-His_6_* gene | Cml | This study |
| pHT1272 | Derivative of pHT1262 carrying a *pamA K246amb-His_6_* gene | Cml | This study |
| pHT1273 | Derivative of pHT1262 carrying a *pamA K273amb-His_6_* gene | Cml | This study |
| pHT1275 | Derivative of pHT1262 carrying a *pamA K284amb-His_6_* gene | Cml | This study |
| pHT1277 | Derivative of pHT1262 carrying a *pamA K331amb-His_6_* gene | Cml | This study |
| pHT1280 | Derivative of pHT1262 carrying a *pamA K356amb-His_6_* gene | Cml | This study |
| pHT1281 | Derivative of pHT1262 carrying a *pamA K371amb-His_6_* gene | Cml | This study |
| pHT1285 | Derivative of pHT1262 carrying a *pamA K407amb-His_6_* gene | Cml | This study |
| pHT1286 | Derivative of pHT1262 carrying a *pamA* *K408amb-His_6_* gene | Cml | This study |
| pHT1287 | Derivative of pHT1262 carrying a *pamA K420amb-His_6_* gene | Cml | This study |
| pHT1288 | Derivative of pHT1262 carrying a *pamA K470amb-His_6_* gene | Cml | This study |
| pHT1289 | Derivative of pHT1262 carrying a *pamA K486amb-His_6_* gene | Cml | This study |
| pHT1290 | Derivative of pHT1262 carrying a *pamA K491amb-His_6_* gene | Cml | This study |
| pHT1291 | Derivative of pHT1262 carrying a *pamA K495amb-His_6_* gene | Cml | This study |
| pHT1292 | Derivative of pHT1262 carrying a *pamA K388amb-His_6_* gene | Cml | This study |
| pHT1293 | Derivative of pHT1262 carrying a *pamA* *K389amb-His_6_* gene | Cml | This study |

Cml stands for a chloramphenicol resistance marker.
