# Supplementary material for: Genetic incorporation of non-canonical amino acid photocrosslinkers in Neisseria meningitidis: New method provides insights into the physiological function of the function-unknown NMB1345 protein
Source: PLoS One. 2020 Aug 31;15(8):e0237883. doi: 10.1371/journal.pone.0237883 (PMC7458321; doi:10.1371/journal.pone.0237883)
Supplement: S5 Table — Pili isolated from N. meningitidis strains HT1125 (wild-type) and HT1822 (ΔpamA) were subjected to trypsin digestion. Labelling of the peptides and analysis by LC-MS/MS were performed as described previously [38]. Proteins were identified by running MASCOT against the NCBI database (NCBInr 20160202). Ratio of the protein levels were expressed as the value of relative amount in HT1822 divided into those in HT1125. The results from four independent experiments were shown in this table. (DOCX) [file pone.0237883.s018.docx]

**S5 Table Analysis of Pili components by Tandem Mass Tag labeling**

|  |  |  | *ΔpamA* / *wild-type* | | | |
| --- | --- | --- | --- | --- | --- | --- |
| Accession No. | Description | Component | Exp.1 | Exp.2 | Exp.3 | Exp.4 |
| 488164107 | pilin [Neisseria meningitidis] | **PilE** | 0.017 |  | 0.022 |  |
| 401664182 | fimbrial protein [Neisseria meningitidis] | **PilE** | 0.039 | 0.052 | 0.030 | 0.039 |
| 496712676 | fimbrial protein [Neisseria meningitidis] | **PilE** | 0.060 |  |  |  |
| 389606015 | type IV pilus assembly protein PilX [Neisseria meningitidis alpha522] | PilX | 0.118 | 0.073 | 0.047 | 0.066 |
| 254672032 | truncated pilin, partial [Neisseria meningitidis alpha275] | **PilE** | 0.124 | 0.207 | 0.032 | 0.211 |
| 488165356 | pilS cassette [Neisseria meningitidis] | **PilE** | 0.128 | 0.171 | 0.188 | 0.169 |
| 488182346 | type IV pilus modification protein PilV [Neisseria meningitidis] | PilV | 0.154 |  |  |  |
| 488165681 | pilus assembly protein PilV [Neisseria meningitidis] | PilV | 0.184 |  |  |  |
| 372202591 | prepilin-type N-terminal cleavage/methylation domain protein [Neisseria meningitidis NM233] | **PilE** | 0.192 | 0.152 | 0.307 | 0.320 |
| 488169696 | pilS cassette [Neisseria meningitidis] | **PilE** | 0.218 | 0.178 |  | 0.159 |
| 639155844 | fimbrial protein [Neisseria meningitidis] | **PilE** | 0.289 |  |  |  |
| 488169551 | pilus biosynthesis protein PilC [Neisseria meningitidis] | PilC | 3.166 | 4.397 |  | 3.464 |
| 485393131 | pilin family protein, partial [Neisseria meningitidis 2003051] | **PilE** | 6.552 |  |  |  |
| 325128394 | type IV pilus biogenesis/stability protein [Neisseria meningitidis N1568] | PilF | 14.645 | 32.856 | 2.530 |  |
| 488179143 | type IV pilus assembly PilM family protein [Neisseria meningitidis] | PilM | 21.461 |  |  |  |
| 488183700 | pilus assembly, PilO family protein [Neisseria meningitidis] | PilO | 21.694 | 86.193 |  |  |
| 754507050 | pilus assembly protein [Neisseria meningitidis] | PilC | 29.766 |  |  | 31.532 |
| 488143312 | pilus assembly protein PilP [Neisseria meningitidis] | PilP | 29.888 |  |  |  |
