# Supplementary material for: Genetic incorporation of non-canonical amino acid photocrosslinkers in Neisseria meningitidis: New method provides insights into the physiological function of the function-unknown NMB1345 protein
Source: PLoS One. 2020 Aug 31;15(8):e0237883. doi: 10.1371/journal.pone.0237883 (PMC7458321; doi:10.1371/journal.pone.0237883)
Supplement: S1 File — (PDF) [file pone.0237883.s019.pdf]

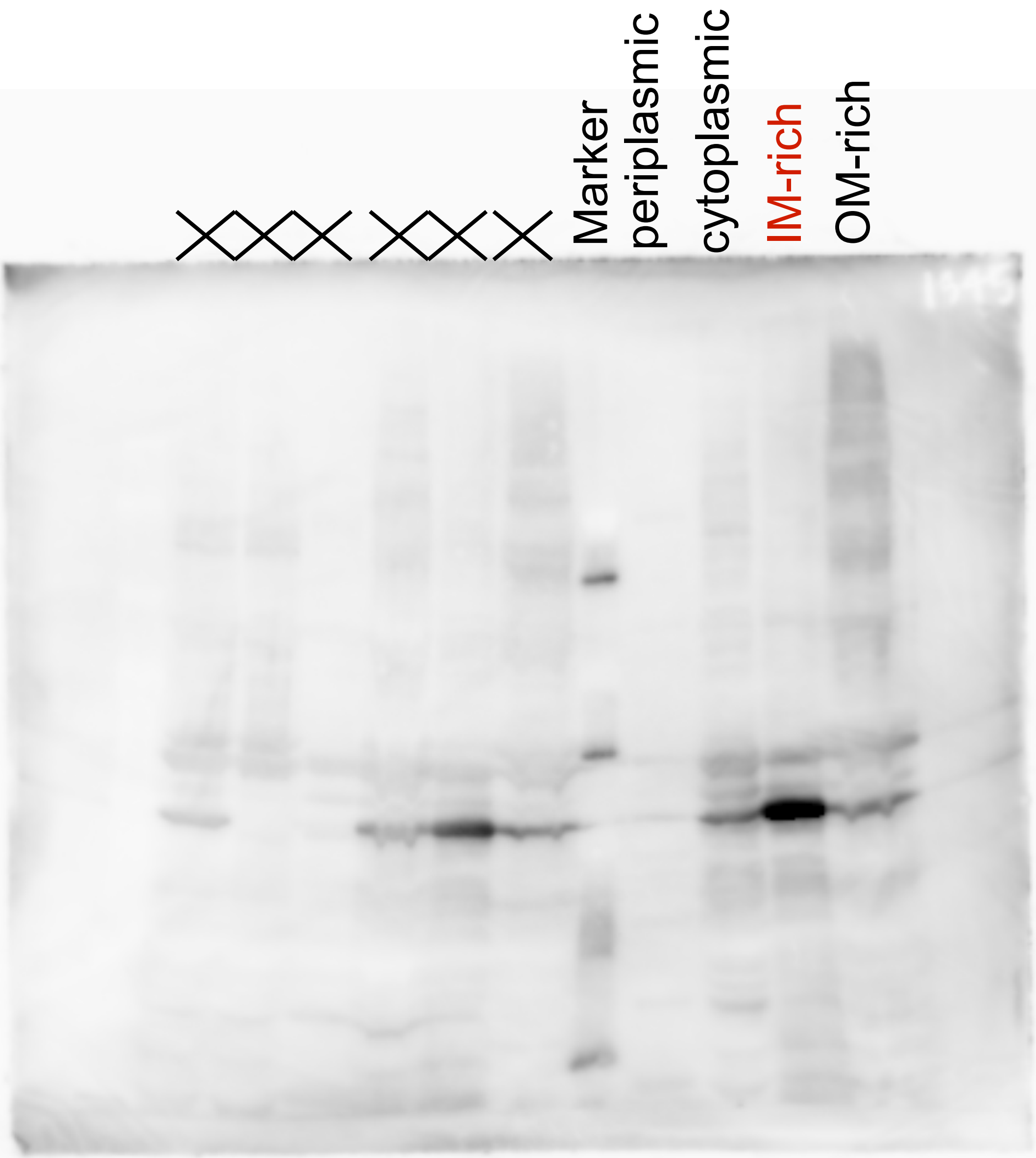


@ Detected by anti -PamA rabbit serum & anti-rabbit-HRP conjugate-2nd antibody

@ Captured by Chemiliminace imaging system: FUSION SOLO 5 (M&S)

@ Use by cut out left-half of the gel to show the fractionation of PamA protein in *N .meningitidis*

Fig 2A_raw_image

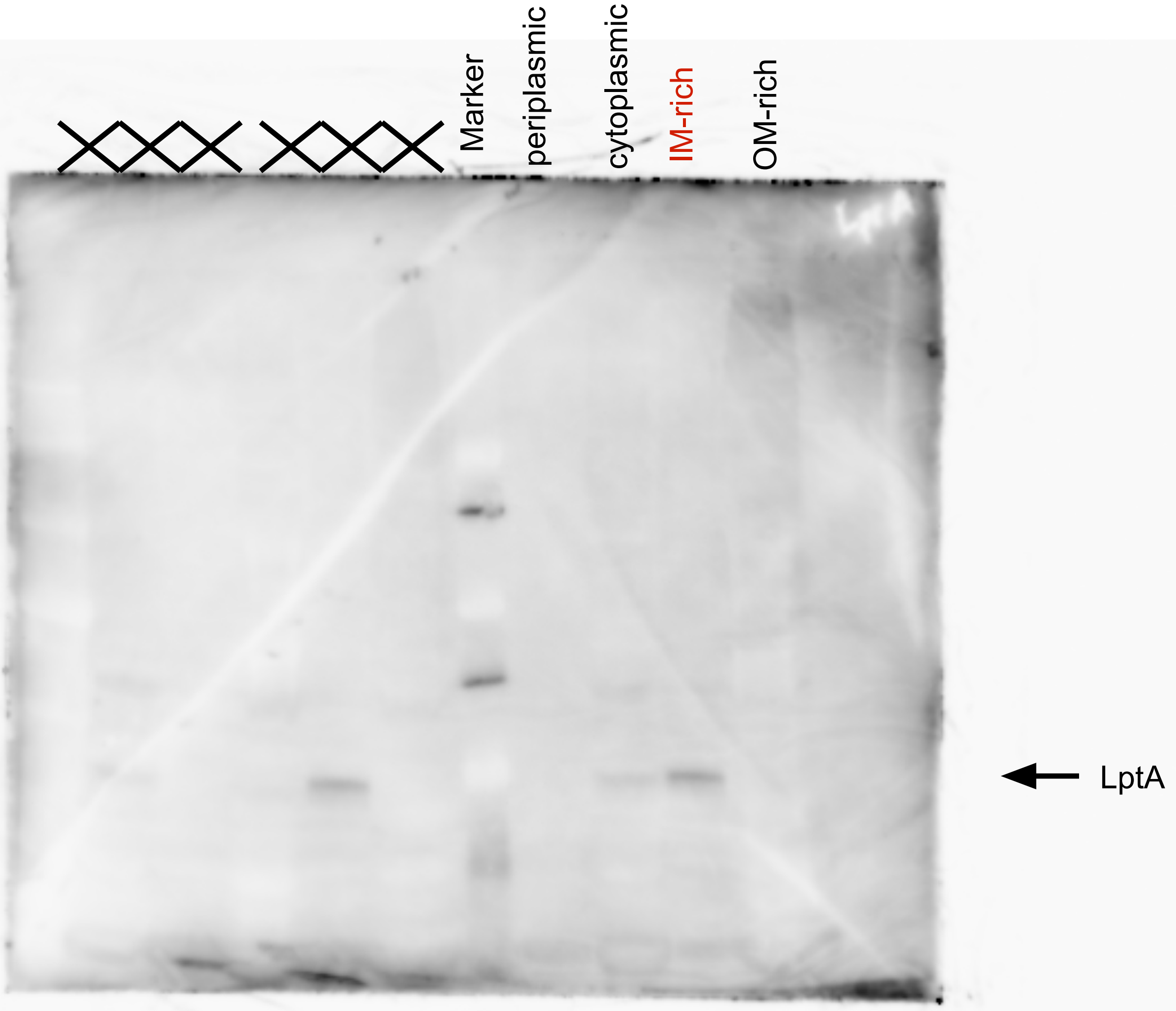


@ Detected by anti-LptA rabbit serum & anti-rabbit-HRP conjugate-2nd antibody

@ Captured by Chemiliminace imaging system: FUSION SOLO 5 (M&S)

@ Use by cut out right-half of the gel to show the fractionation of LptA protein
(inner membrane protein) in *N .meningitidis*

Fig 2A-2_raw_image

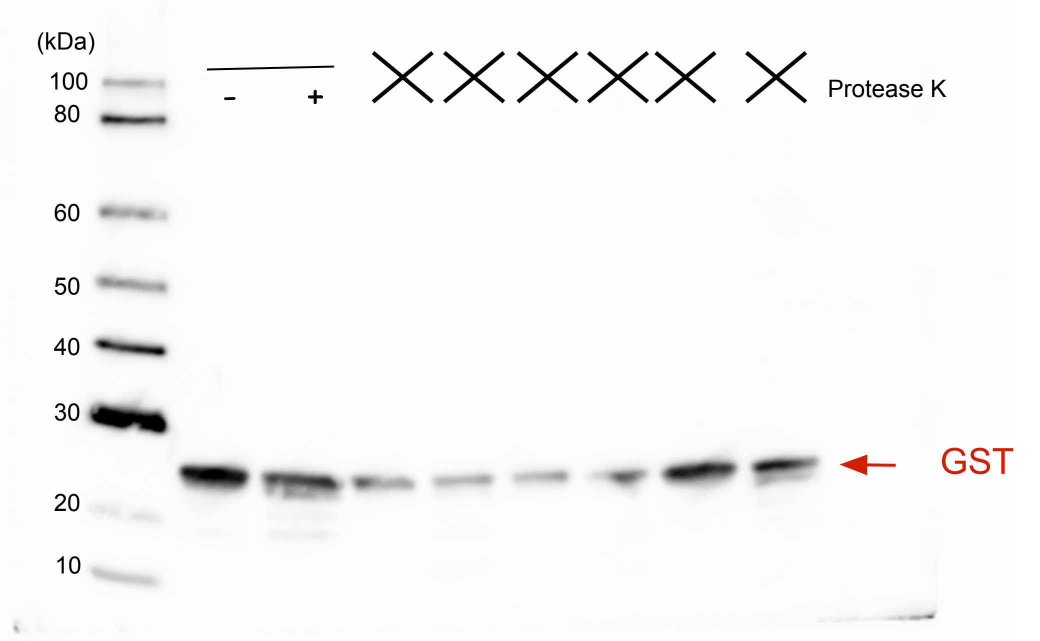


@ Detected meningocccal spheroplasted  meningococcil digested by Protease K
followed by SDS-PAGE & Western blotting

@ Detectedby anti -GST mAb & anti-mouse-HRP conjugate-2nd antibody

@ Captured by Chemiliminace imaging system: FUSION SOLO 5 (M&S)

@ Use by cutting out the left part of bands to show the digestion of GST protein in *N .meningitidis*

Fig 2D_raw_image

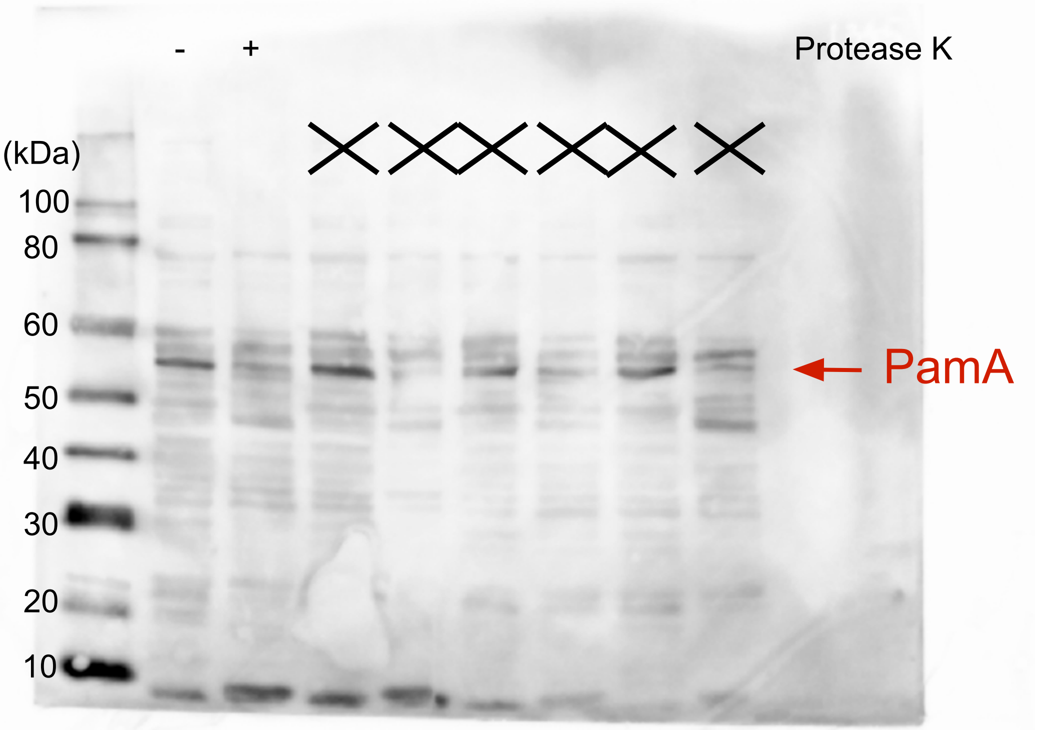


@ Detected spheroplasted meningococci digested by Protease K
followed by SDS-PAGE & Western blotting

@ Detected by anti-PamA rabbit serum & anti-rabbit-HRP conjugate-2nd antibody

@ Captured by Chemiliminace imaging system: FUSION SOLO 5 (M&S)

@ Use by cutting out the left part of the image to show the digestion of PamA protein

Fig 2E_raw_image

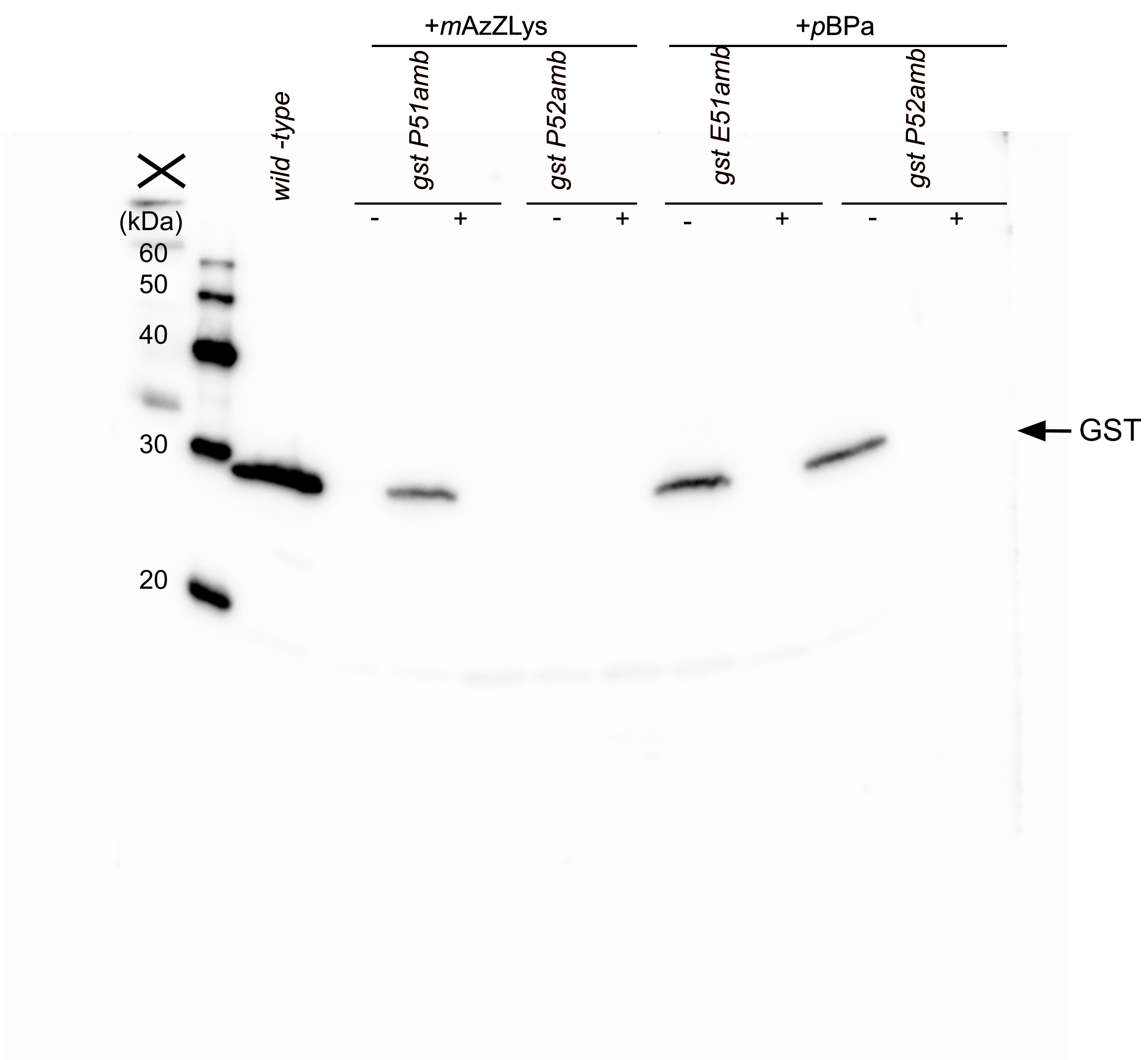


@ Effeciency of incorporation of ncAAs into *gst P51amb* or *P52amb* against wt GST by SDS-PAGE & Western blotting

@ Detected by anti-GST mAb & anti-mouse-HRP conjugate-2nd antibody

@ Captured by Chemiliminace imaging system: FUSION SOLO 5 (M&S)

@ Used by cutting out to remove margin of the image

Fig 3B_raw_image

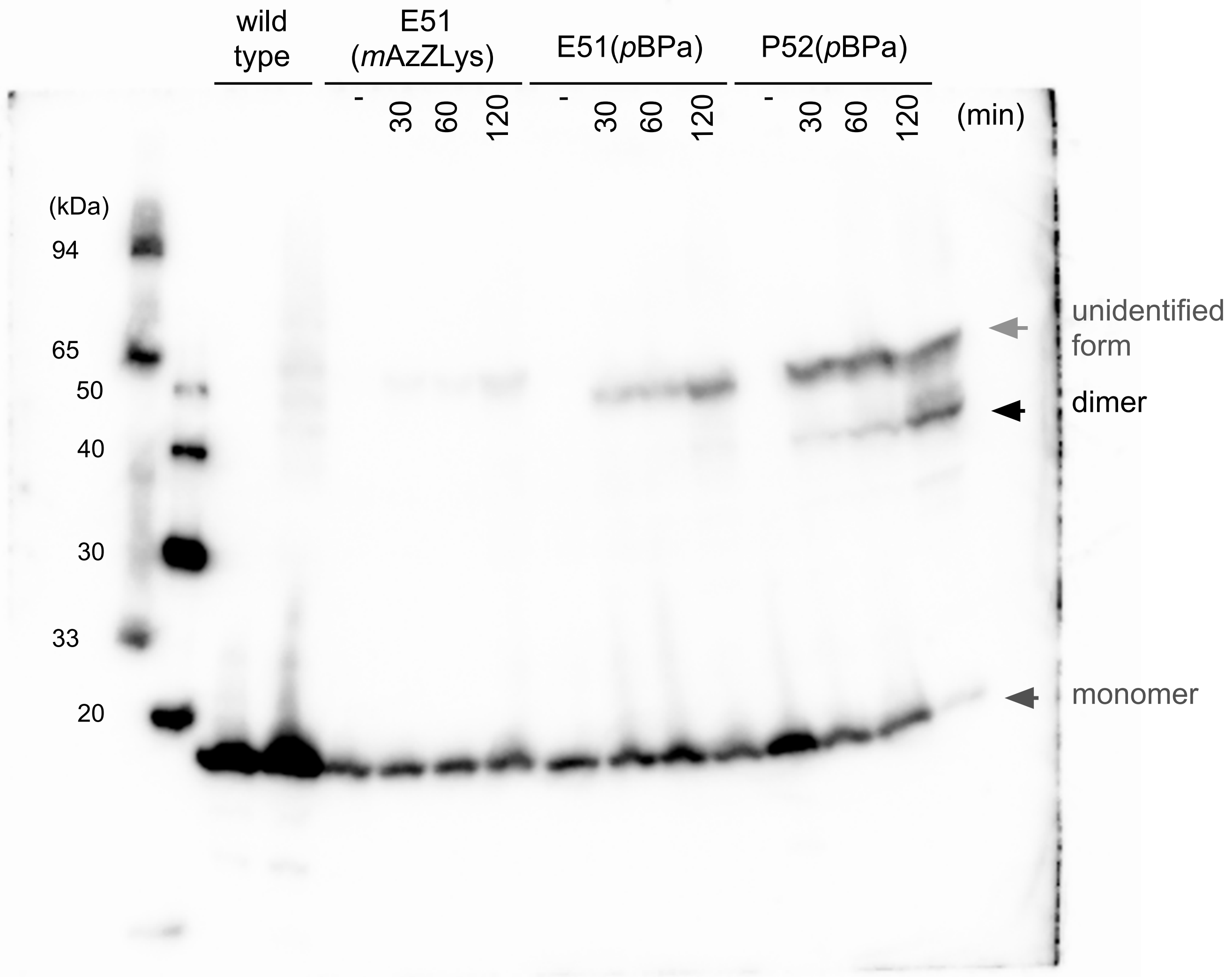


@ Effeciency of crosslinking with ncAAs in GST E51amb  and P52amb monitored by Western blotting

@ Detected by anti-GST mAb & anti-mouse-HRP conjugate-2nd antibody

@ Captured by Chemiliminace imaging system: FUSION SOLO 5 (M&S)

@ Used by cutting out margin of the image

Fig 3C_raw_image

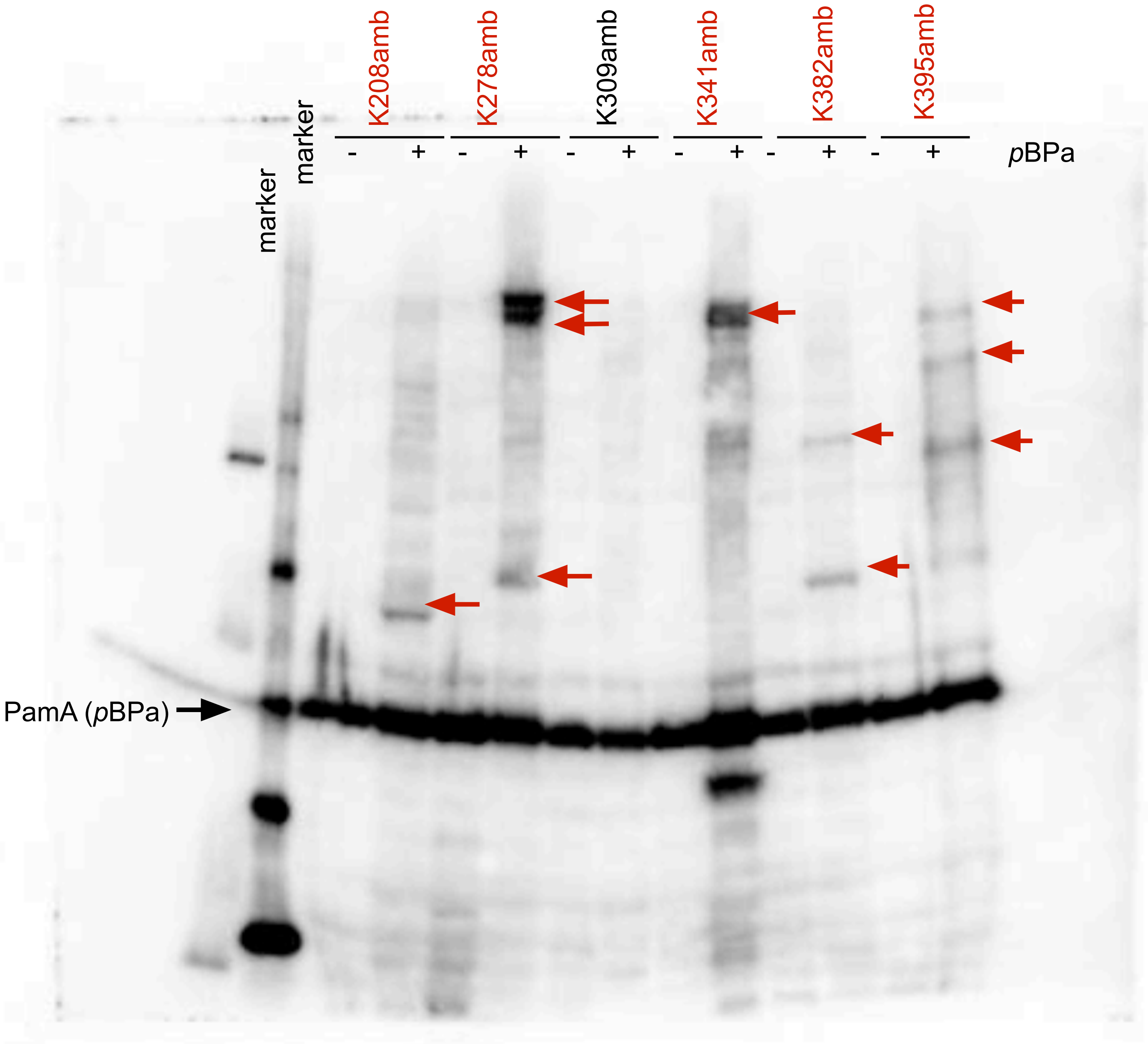


bands crosslinked with PamA (*p*BPa)

@ re-examination of samples used in S3 Fig by SDS-PAGE & Western blotting

@ Detected by anti -(His)6 mAb & anti-mouse-HRP conjugate-2nd antibody

@ Captured by Chemiliminace imaging system: FUSION SOLO 5 (M&S)

@ Used this panel without any modification

Fig 4_raw_image

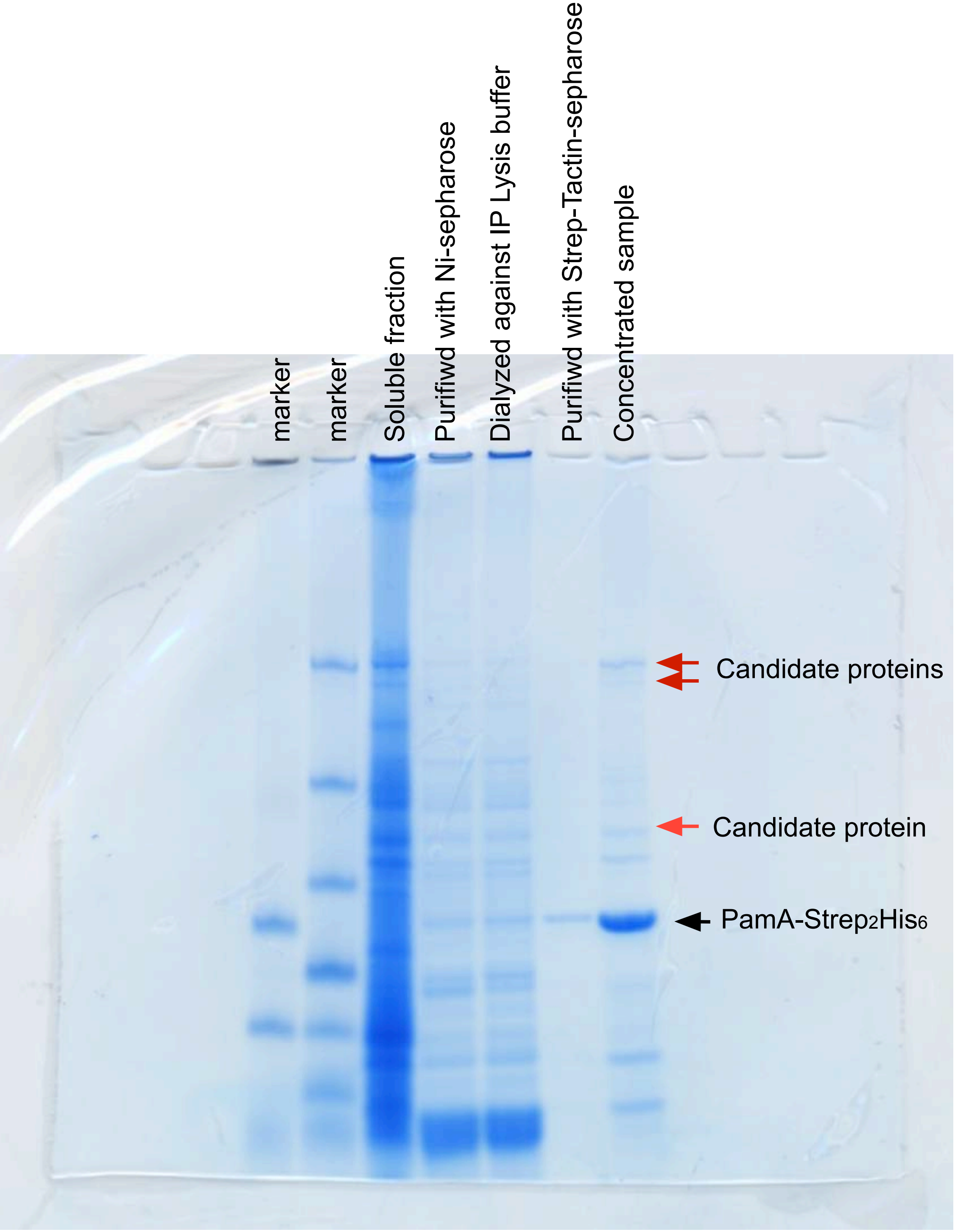


@ Samples purified with His resin & StrepTactin resin and analyzed by SDS-PAGE

@ Visulalized by CBB staining

@ captured by a general scanner

@ Used by cutting out the center of the image

Fig 5A_raw_image

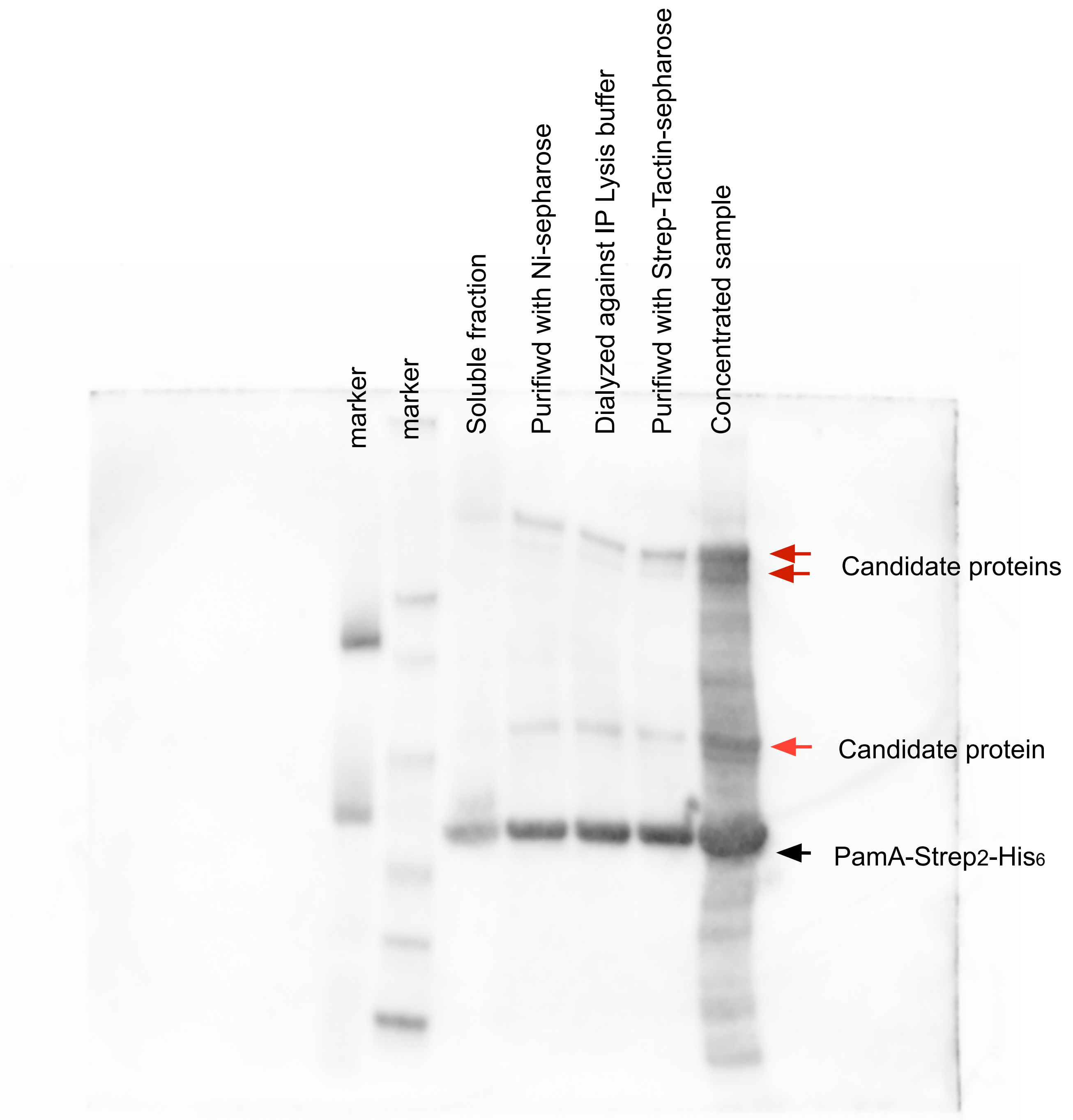


@ Purified samples with His resin & StrepTaction by SDS-PAGE & Western blotting

@ Detectedby anti-(His)6 mAb & anti-mouse-HRP conjugate-2nd antibody

@ Captured by Chemiliminace imaging system: FUSION SOLO 5 (M&S)

@ Used by cutting out the center of the image

Fig 5B_raw_image

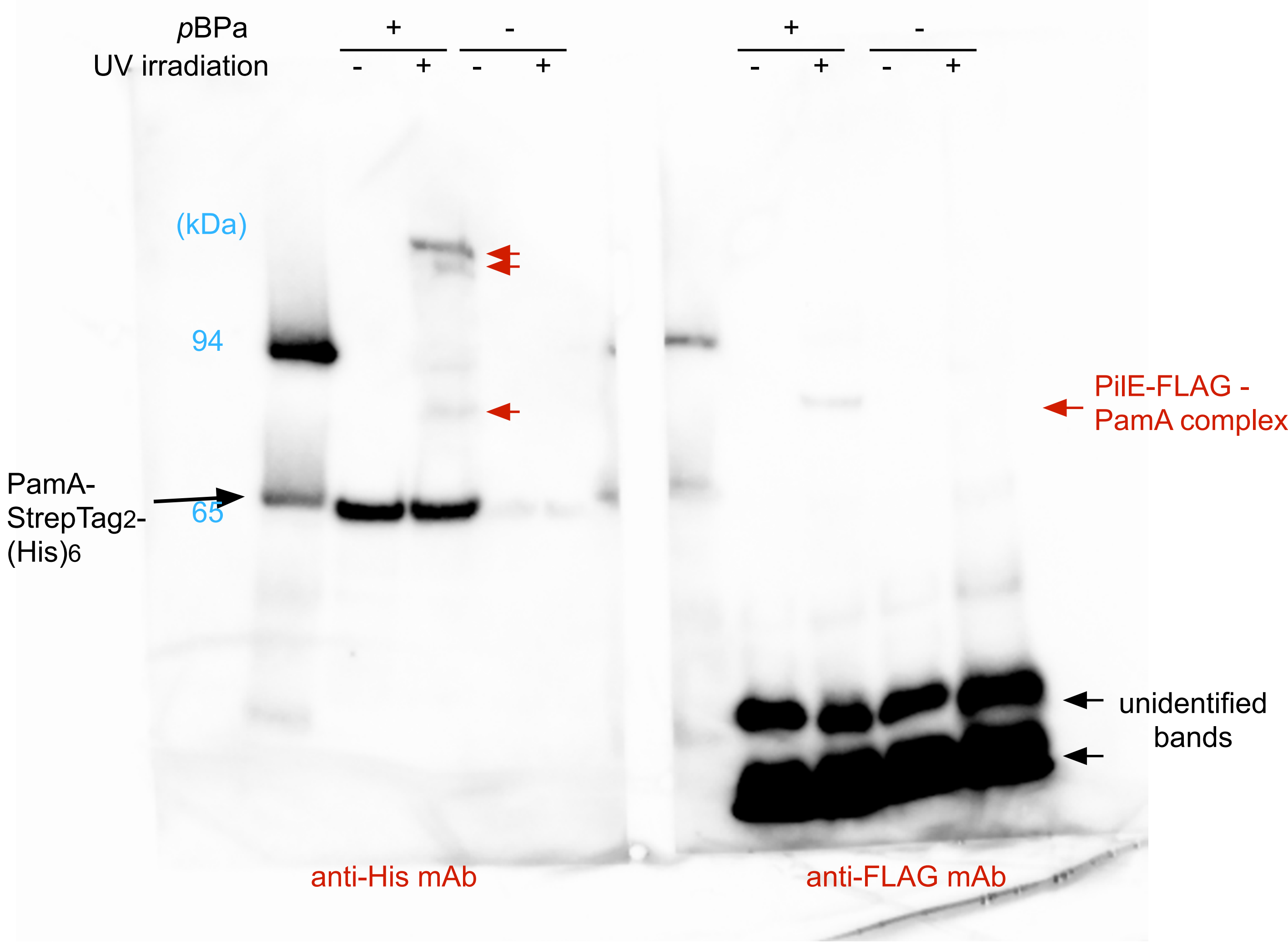


@ Purified crosslinked samples with His resin by SDS-PAGE & Western blotting

@ Detected by anti-$(His)_6$ & FLAG mAbs & anti-mouse-HRP conjugate-2nd antibody

@ Captured by Chemiliminace imaging system: FUSION SOLO 5 (M&S)

@ Used by cutting out the upper part of the image

Fig 5C_raw_image

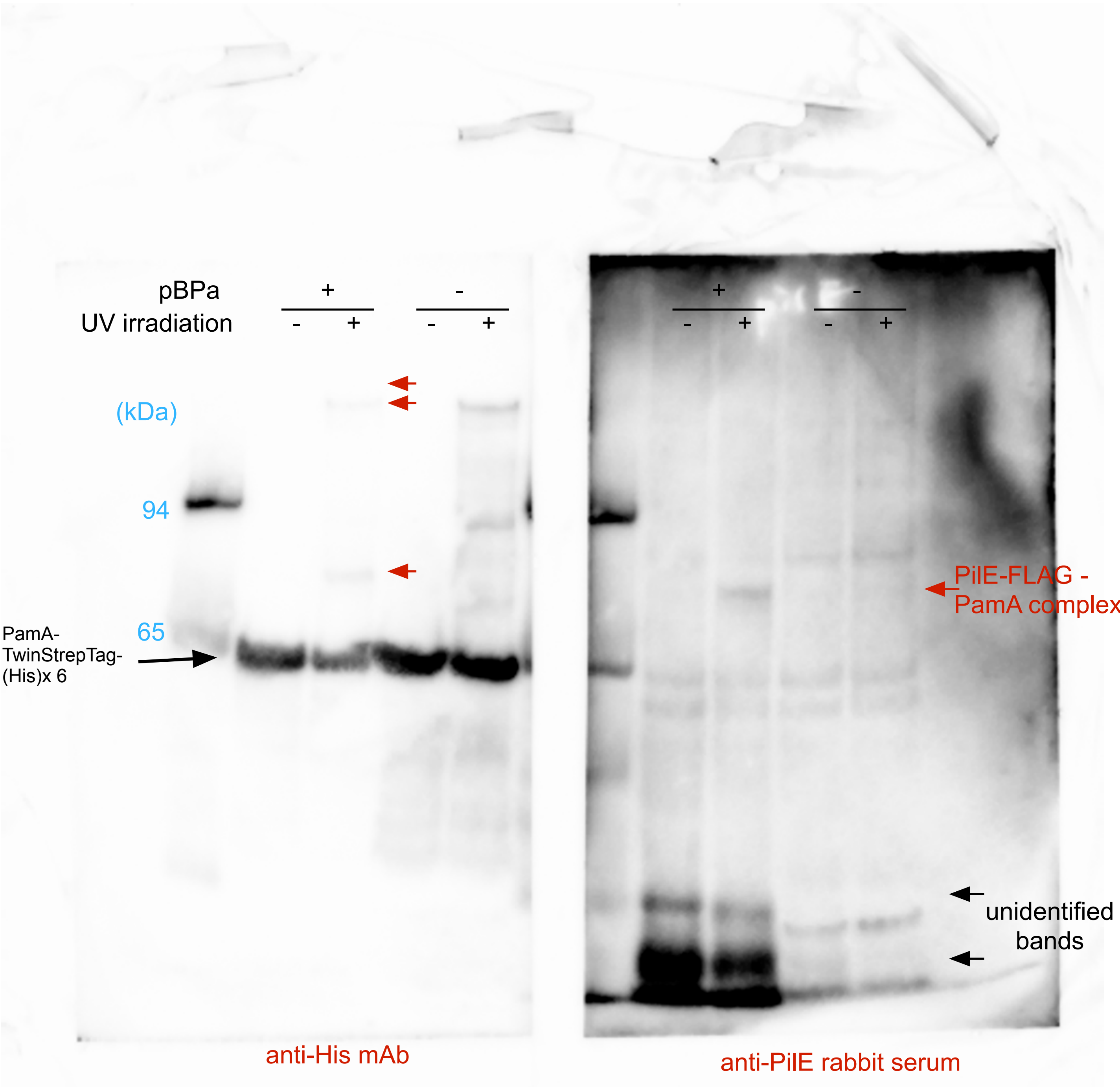


@ Purified crosslinked samples with His resin by SDSP-AGE & Western blotting

@ Detected by anti -(His)6 mAb & anti PilE rabbit serum & anti-mouse/rabbit-HRP conjugate-2nd antibody

@Captured by Chemiliminace imaging system: FUSION SOLO 5 (M&S)

@ Used by cutting out the center of the image

Fig 5D_raw_image

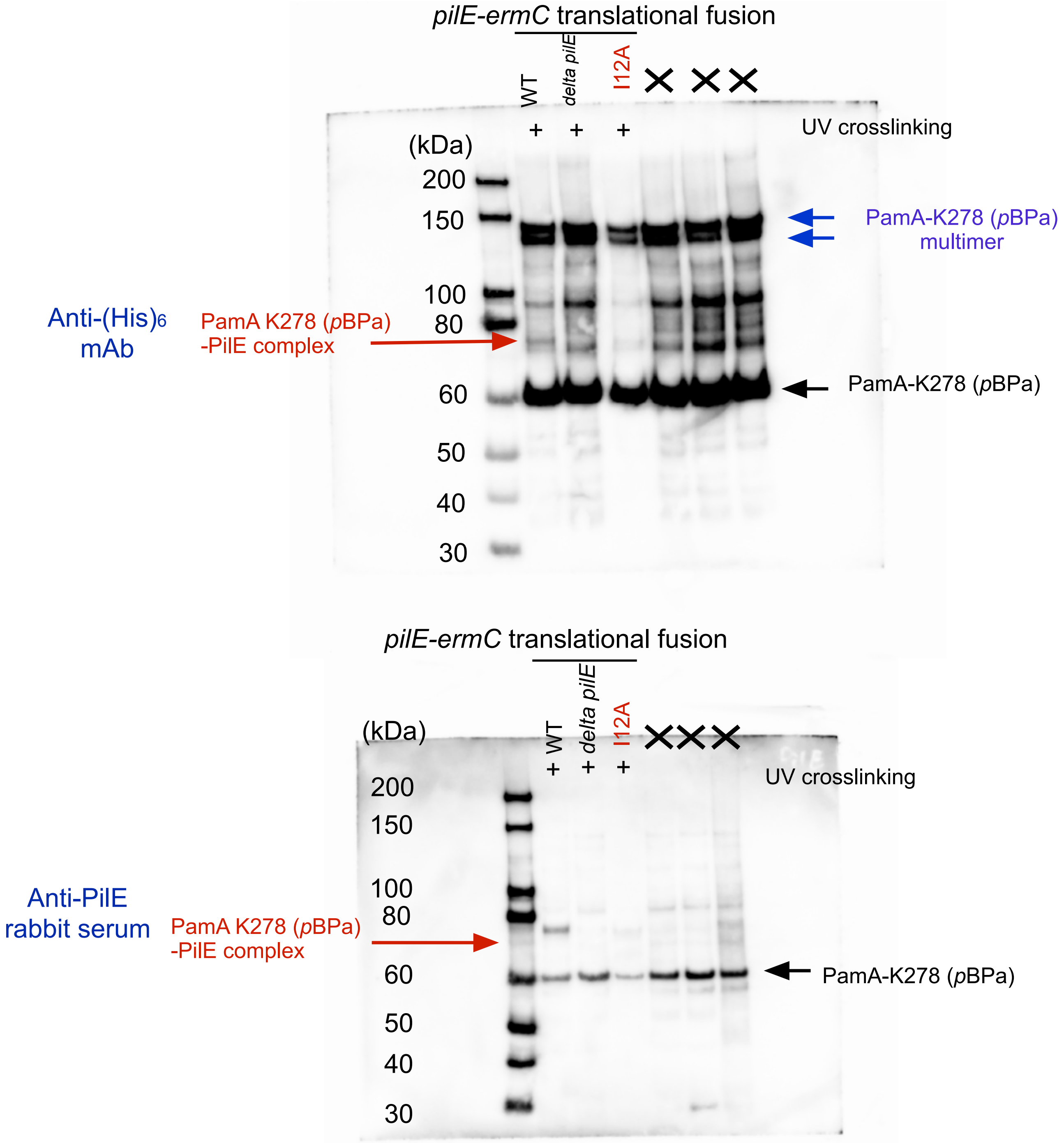


@ Purified crosslinked samples with His resin by SDSP-AGE & Western blotting

@ Detectedby anti-(His)6 mAb & anti PilE rabbit serum & anti-mouse/rabbit-HRP conjugate-2nd antibody

@ Captured by Chemiliminace imaging system: FUSION SOLO 5 (M&S)

@ Used by cutting out the left part of the gel

Fig 6C_raw_image

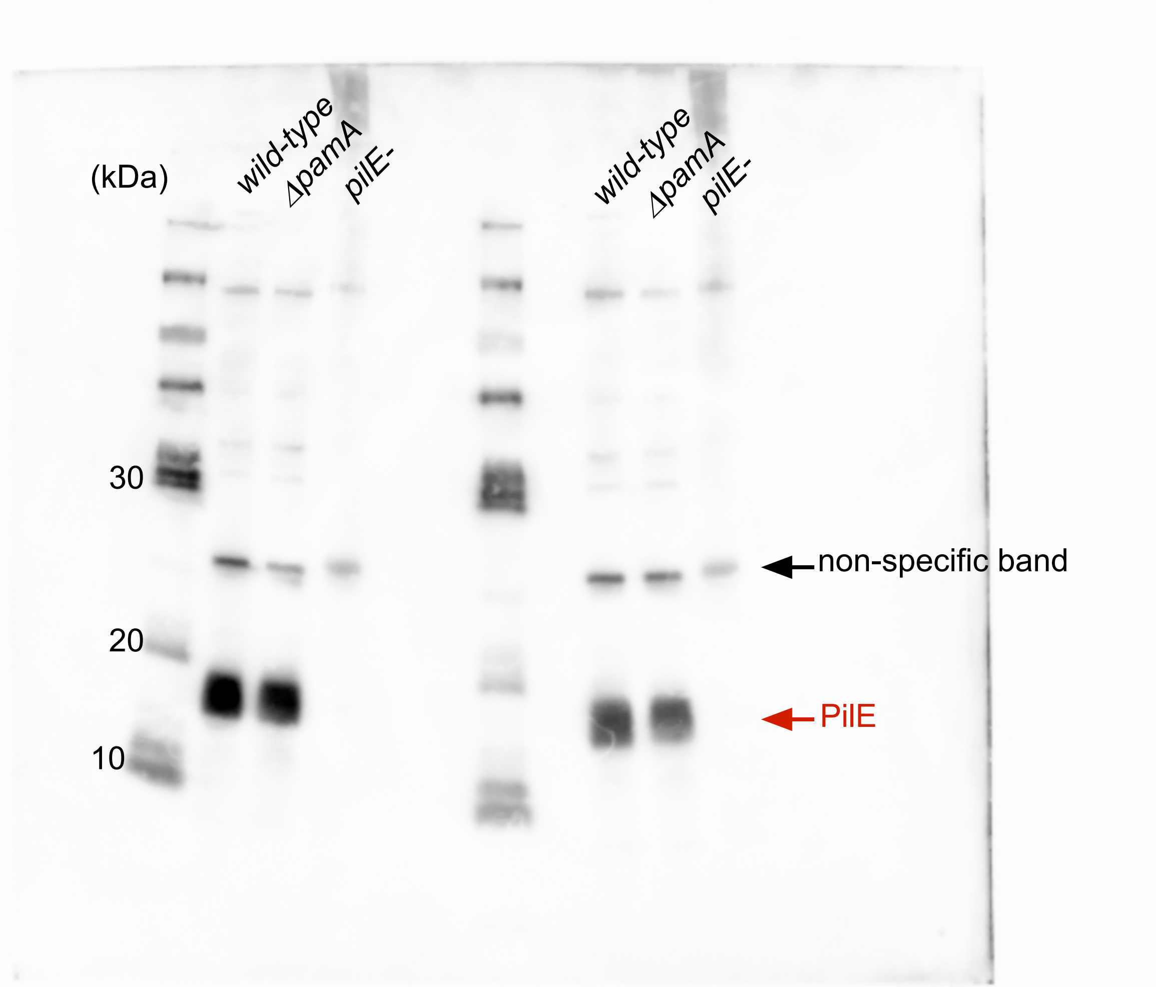


@ PilE expression in these *N. meningitidis* strains by SDS-PAGE & Western blotting
@ Detected by anti-PilE rabbit serum & anti-rabbit-HRP conjugate-2nd antibody
@ Captured by Chemiliminace imaging system: FUSION SOLO 5 (M&S)
@ Used by cutting out the left part of the image

Fig 7B_raw_image

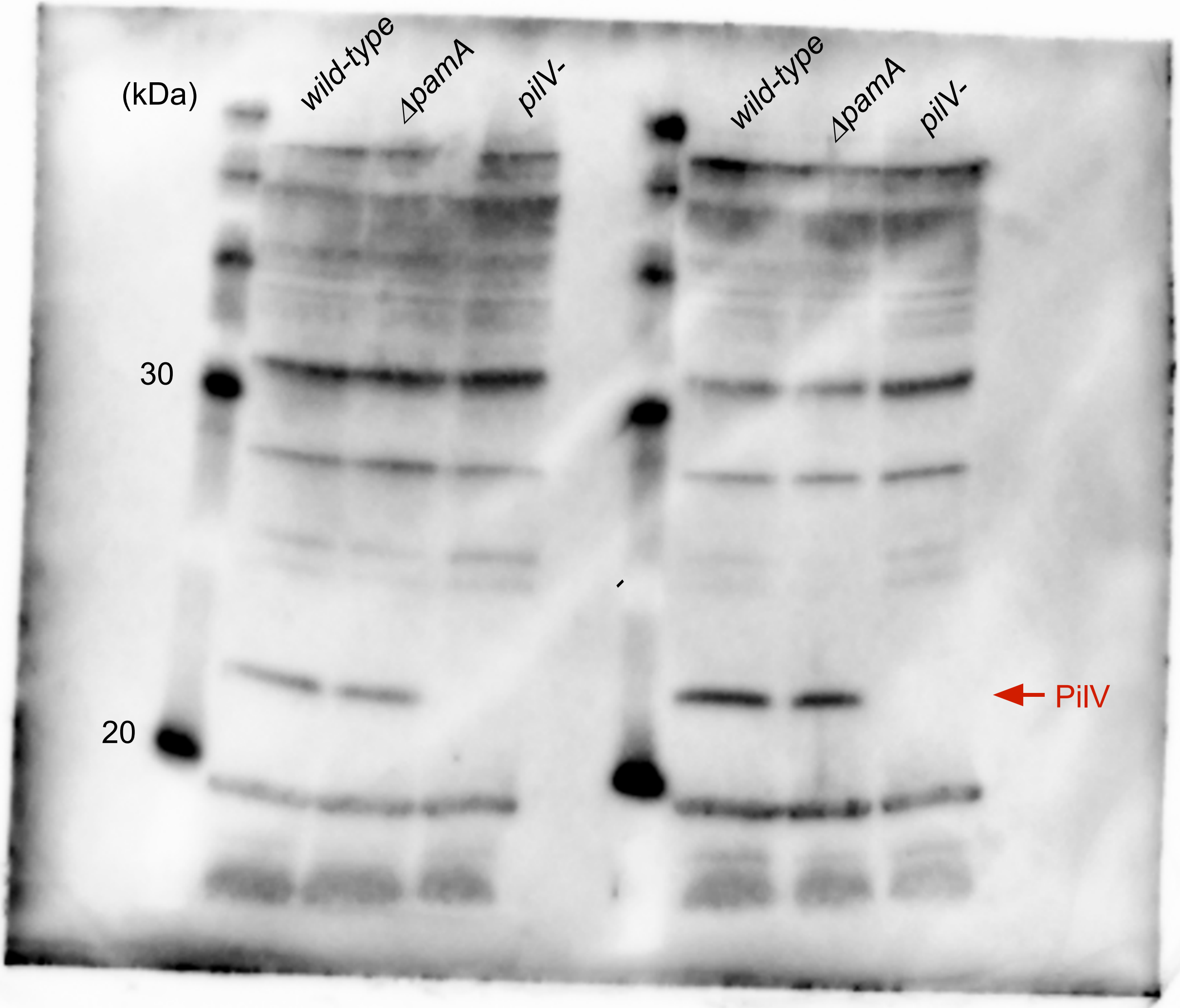


@ PilV expression in these *N. meningitidis* strains by SDS-PAGE & Western blotting
@ Detectedby anti-PilV rabbit serum & anti-rabbit-HRP conjugate-2nd antibody
@ Captured by Chemiliminace imaging system: FUSION SOLO 5 (M&S)
@ Used by cutting out the right part of the image

Fig 7C_raw_image

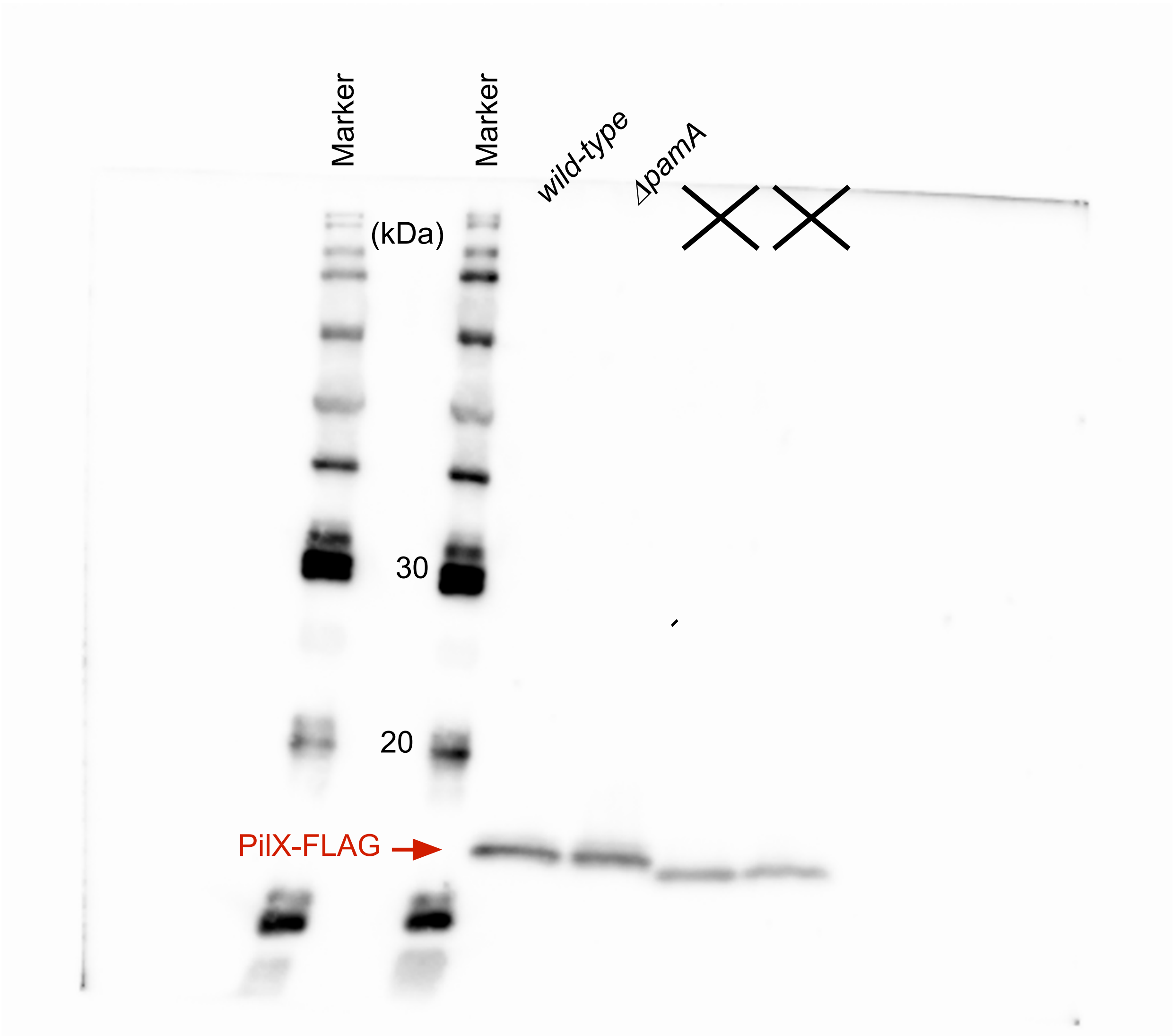


@ PilX-FLAG expression in these *N. meningitidis* strains by SDS-PAGE & Western blotting

@ Detectedby anti-FLAG mAb & anti-mouse-HRP conjugate-2nd antibody

@ Captured by Chemiliminace imaging system: FUSION SOLO 5 (M&S)

@ Used by cutting out the middle part of the image

Fig7 D_raw_image

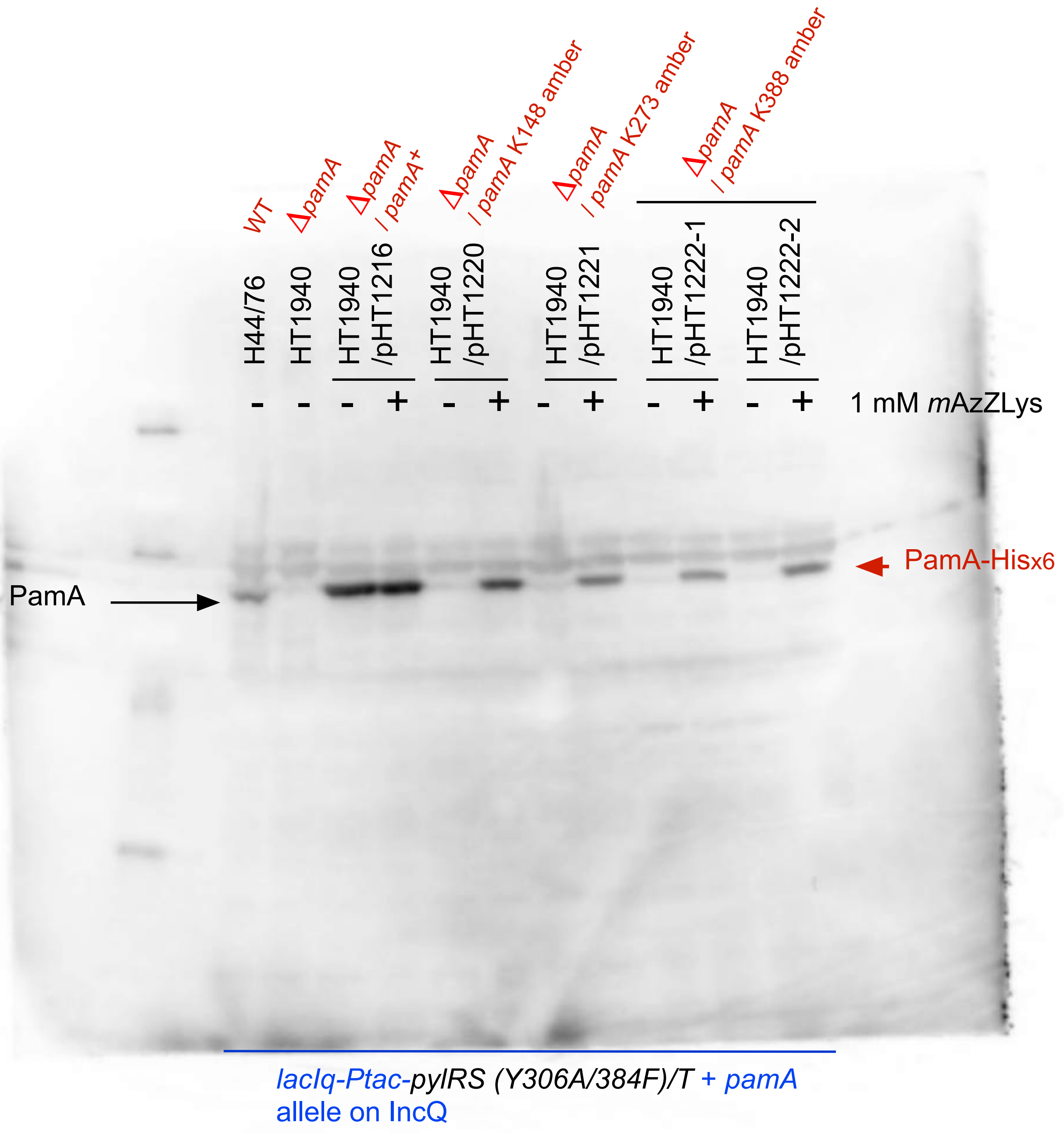


@ *m*AzZLys-dependent expression of PamA amber mutants by SDS-PAGE & Western blotting

@ Detected by anti-PamA rabbit serum & anti-rabbit-HRP conjugate-2nd antibody

@ Captured by Chemiliminace imaging system: FUSION SOLO 5 (M&S)

@ Used by cutting out the left part of the gel

S2A Fig_raw_image

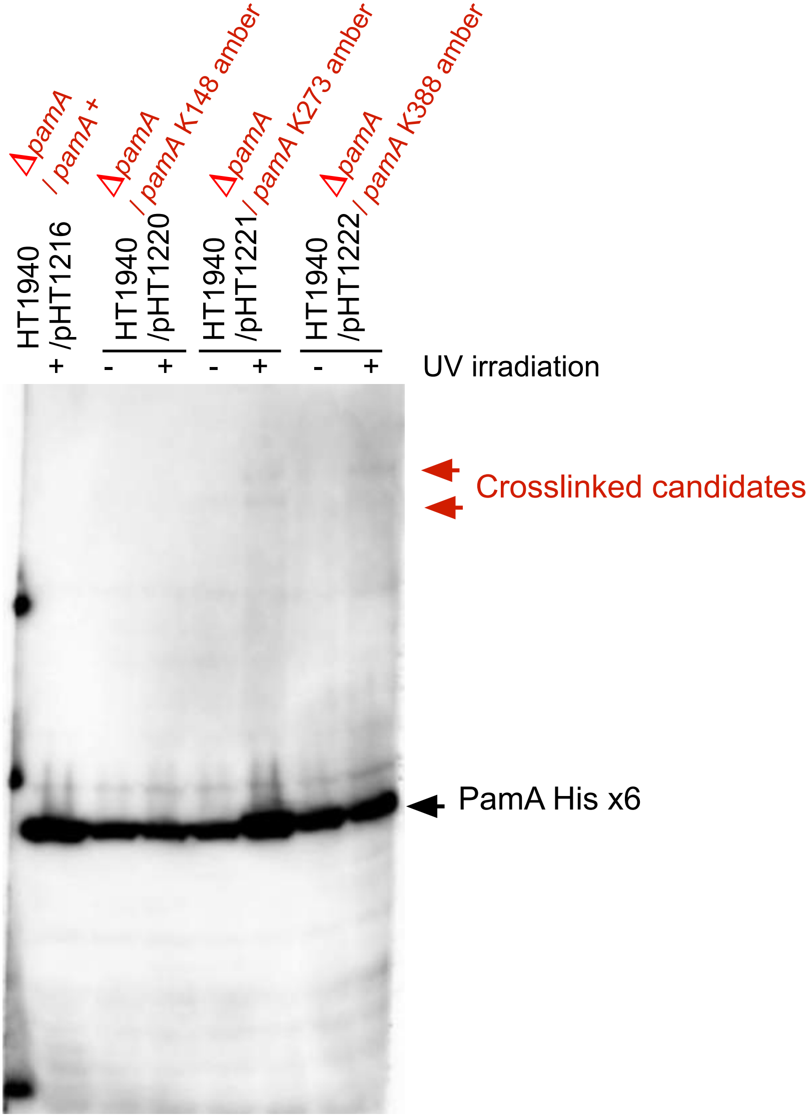


@ UV crosslinked band to the PamA protein containing *mAzZLys* in *N. meningitidis* by SDS-PAGE & Western blotting

@ Detectedby anti-(His)6 mAb & anti-mouse-HRP conjugate-2nd antibody

@ Captured by Chemiliminace imaging system: FUSION SOLO 5 (M&S)

@ Used this image directly

S2B Fig_raw_image

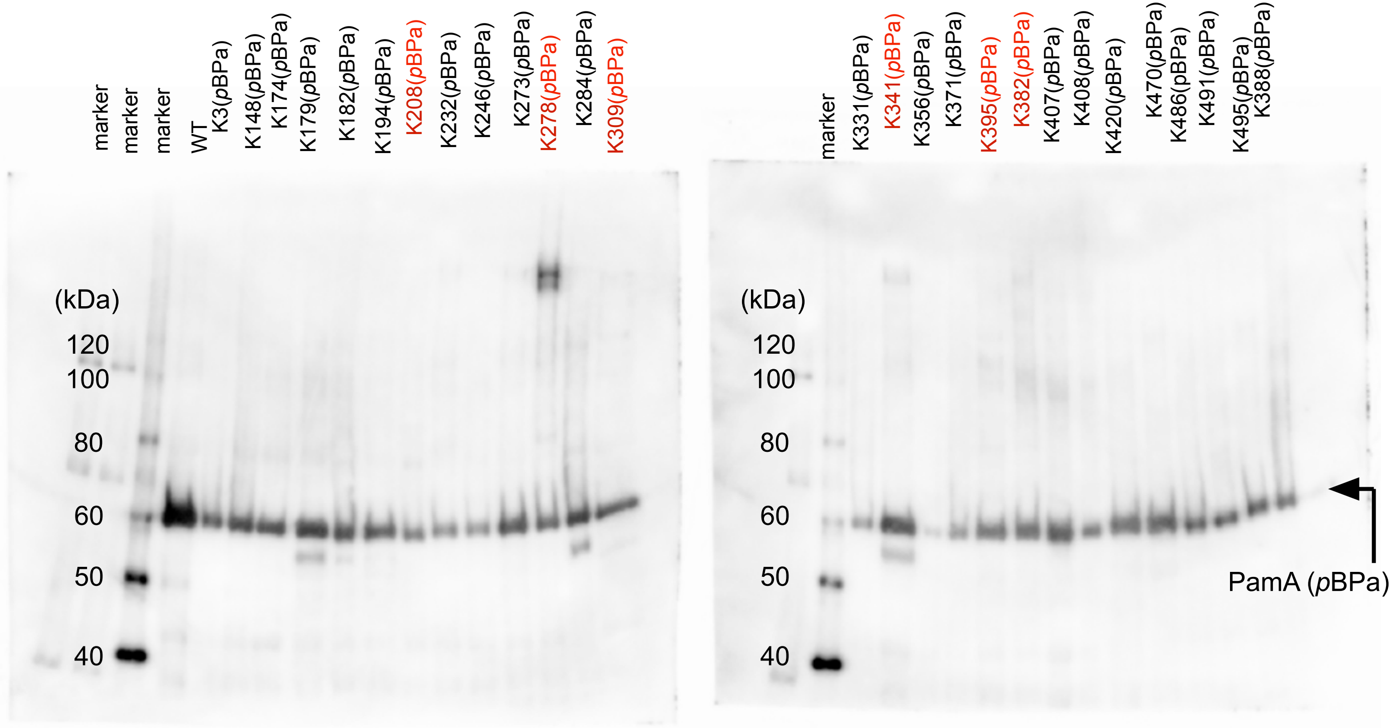


@ Purified samples with His resin & analyzed by SDS-PAGE & Western blotting

@ Detectedby anti-(His)6 mAb & anti-mouse-HRP conjugate-2nd antibody

@ Captured by Chemiliminace imaging system: FUSION SOLO 5 (M&S)

@ Use this image without any moditicilation

S3 Fig_raw_image

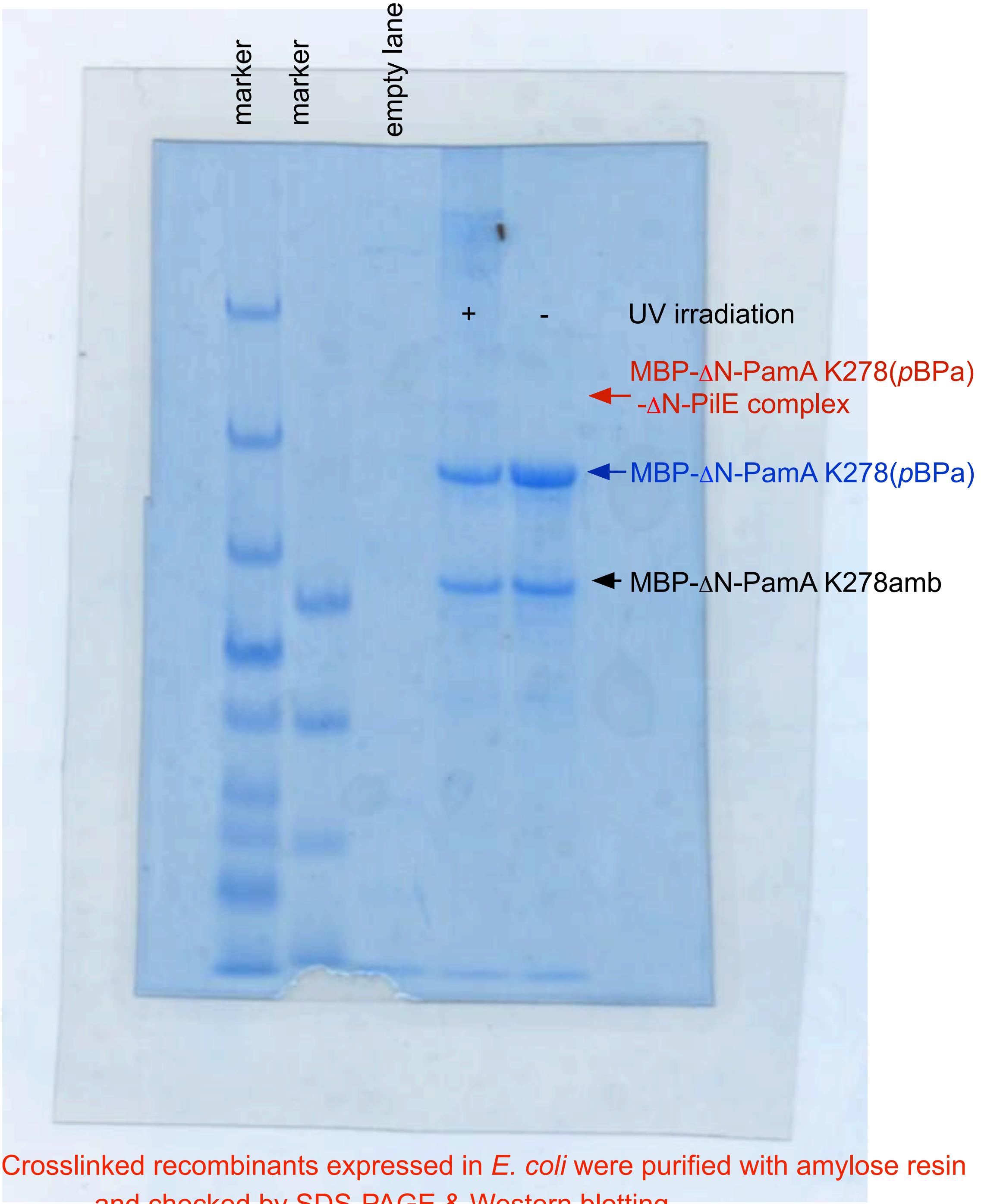


@ Crosslinked recombinants expressed in *E. coli* were purified with amylose resin and checked by SDS-PAGE & Western blotting

@ Detected by staining with CBB

@ Captured by scanner

@ Used by cutting out the left part of the gel

S6A Fig_raw_image

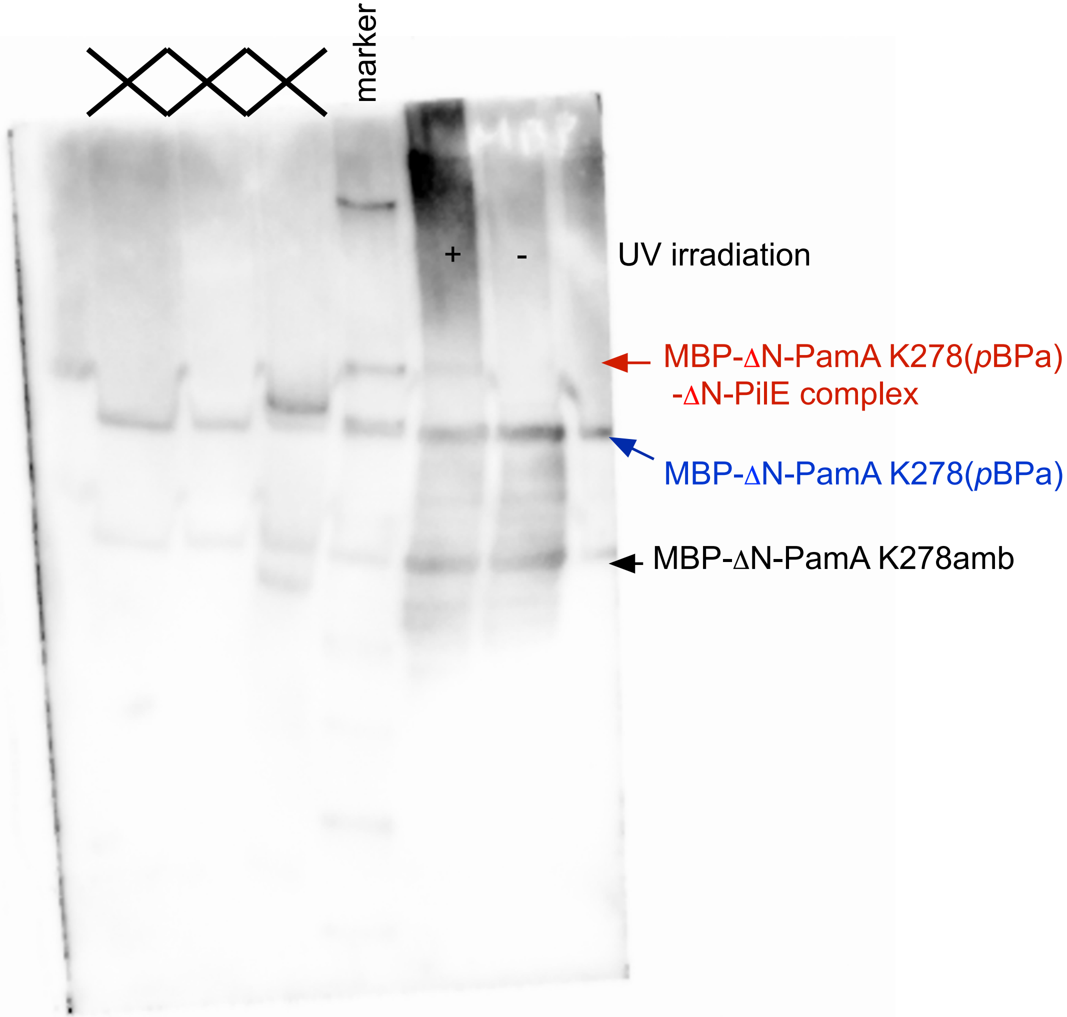


@ Crosslinked recombinants expressed in *E. coli* were purified with amylose resin and checked by SDS-PAGE & Western blotting

@ Detected by staining with anti-MBP mAb & anti-mouse-HRP 2nd antibody

@ Chemiliminace imaging system: FUSION SOLO 5 (M&S)

@ Used by cutting out the right part of the image

S6B Fig_raw_image

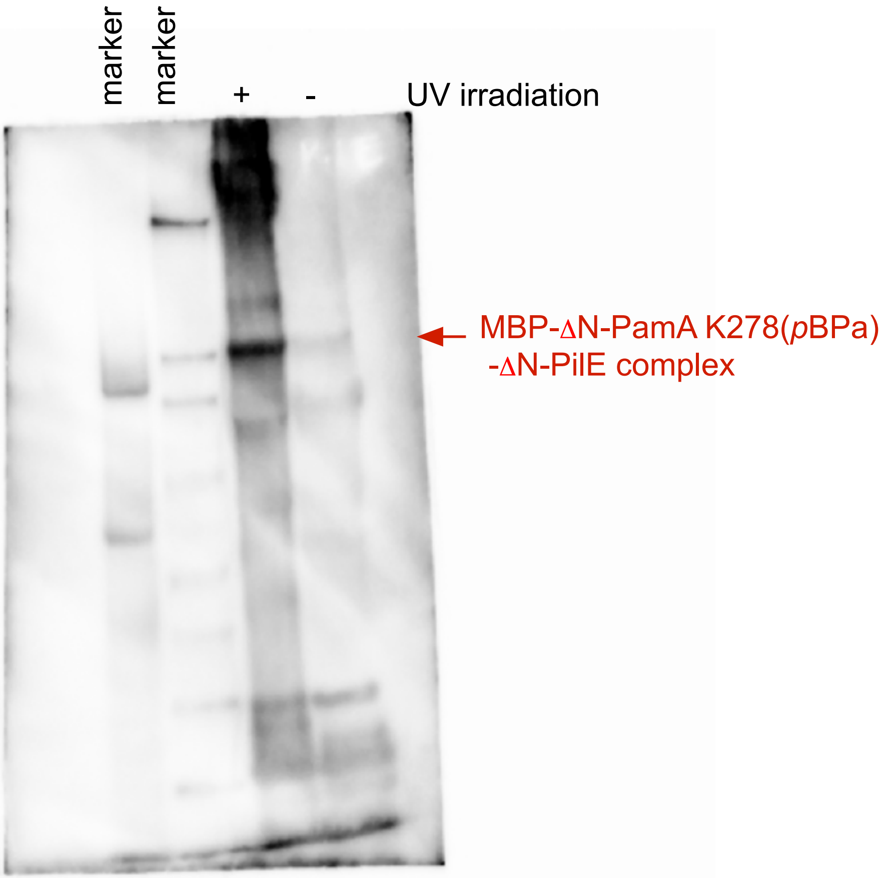


@ Crosslinked protein recombinants expressed in *E. coli* were purified with amylose resin and checked by SDS-PAGE & Western blotting

@ Detected with ant-PilE rabbit serum & anti-rabbit-HRP 2nd antibody

@ Chemiliminace imaging system: FUSION SOLO 5 (M&S)

@ Used  the entire image

S6C Fig_raw_image
